# Microenvironment-Driven 3-Dimensional Biofabrication Enables Sequential Maturation of the Blood–Brain Barrier

**DOI:** 10.34133/research.1408

**Published:** 2026-10-05

**Authors:** Sen Wang, Xinggang Mao, Chengyao Wang, Yingjie Liu, Chenrui Zhang, Siqi Yao, Luge Bai, Joaquim Miguel Oliveira, Rui L. Reis, Ya Bai, Jiankang He, Zhilu Yang, Dichen Li, Ling Wang

**Affiliations:** ^1^ State Key Laboratory for Manufacturing System Engineering, School of Mechanical Engineering, Xi’an Jiaotong University, Xi’an, China.; ^2^ National Innovation Platform (Center) for Industry-Education Integration of Medical Technology, Xi’an Jiaotong University, Xi’an, China.; ^3^ Department of Neurosurgery, Xijing Hospital, Fourth Military Medical University, Xi’an, China.; ^4^ Department of Endocrinology, Genome Institute, The First Affiliated Hospital of Xi’an Jiaotong University, Xi’an, China.; ^5^ 3B’s Research Group, I3Bs-Research Institute on Biomaterials, Biodegradables and Biomimetics, University of Minho, Guimarães, Portugal.; ^6^ ICVS/3B’s-PT Government Associate Laboratory, Braga/Guimarães, Portugal.; ^7^ Department of Neurology, Xijing Hospital, Fourth Military Medical University, Xi’an, China.; ^8^ The Tenth Affiliated Hospital, Southern Medical University (Dongguan People’s Hospital), Dongguan, China.

## Abstract

Three-dimensional (3D) in vitro blood–brain barrier (BBB) models are essential for studying neurovascular development, drug permeability, and disease mechanisms, yet most fail to recapitulate the temporal sequence of cellular maturation. Here, we present a spatiotemporally organized 3D BBB model engineered via dynamic microenvironmental regulation. Temporally controlled enzymatic modulation of an alginate/collagen dual-network hydrogel enabled transition in matrix properties from strong to weak mechanical confinement, thereby triggering the embedded cells to shift from a quiescent to an active state and further enabling the construction of a BBB model with sequential endothelial and supporting cell development. The resulting 3D BBB model exhibits improved barrier integrity, reduced macromolecular leakage, and physiologically relevant drug-selective permeability. This strategy demonstrates that temporally controlled microenvironmental cues can guide multicellular tissue development, providing a versatile platform for neurovascular research and central-nervous-system-targeted drug evaluation.

## Introduction

Three-dimensional (3D) tissue and organ models have become essential in vitro tools for studying development, disease mechanisms, and therapeutic responses. By mimicking the structural, biochemical, and mechanical complexity of native tissues, these models enable advances in personalized medicine, drug screening, and regenerative strategies [1–6]. Recognizing their potential, regulatory agencies such as the US Food and Drug Administration have begun to adopt organ-on-chip and advanced in vitro models as alternatives to animal models, aiming to accelerate drug development while improving predictive accuracy. However, many existing 3D tissue and organ models still fail to capture the spatiotemporally coordinated activation and differentiation processes that are fundamental to tissue morphogenesis [7–10]. That limitation largely arises from fabrication strategies that remain static, fixing matrix properties and cellular arrangements at the time of construction and lacking the ability to evolve with developmental progression.

Among functional barrier tissues, the blood–brain barrier (BBB) is a highly regulated, multilayered vascular interface [11–13] composed primarily of brain microvascular endothelial cells, pericytes, and astrocytes (Fig. 1A). Endothelial cells line the vessel lumen, forming tight junctions that regulate molecular transport; pericytes are embedded within the basement membrane, supporting vascular stability and remodeling; astrocytes form an outer layer, regulating ion balance and neuronal interactions. The BBB ensures highly selective permeability, immune surveillance, and neurovascular coupling. However, most therapeutic compounds cannot effectively cross the BBB, posing a major challenge for central nervous system (CNS) drug delivery. This limitation underscores the importance of physiologically relevant in vitro BBB models for the efficiency of predicting drug permeability and assessing therapeutic efficacy [14].

**Fig. 1.**
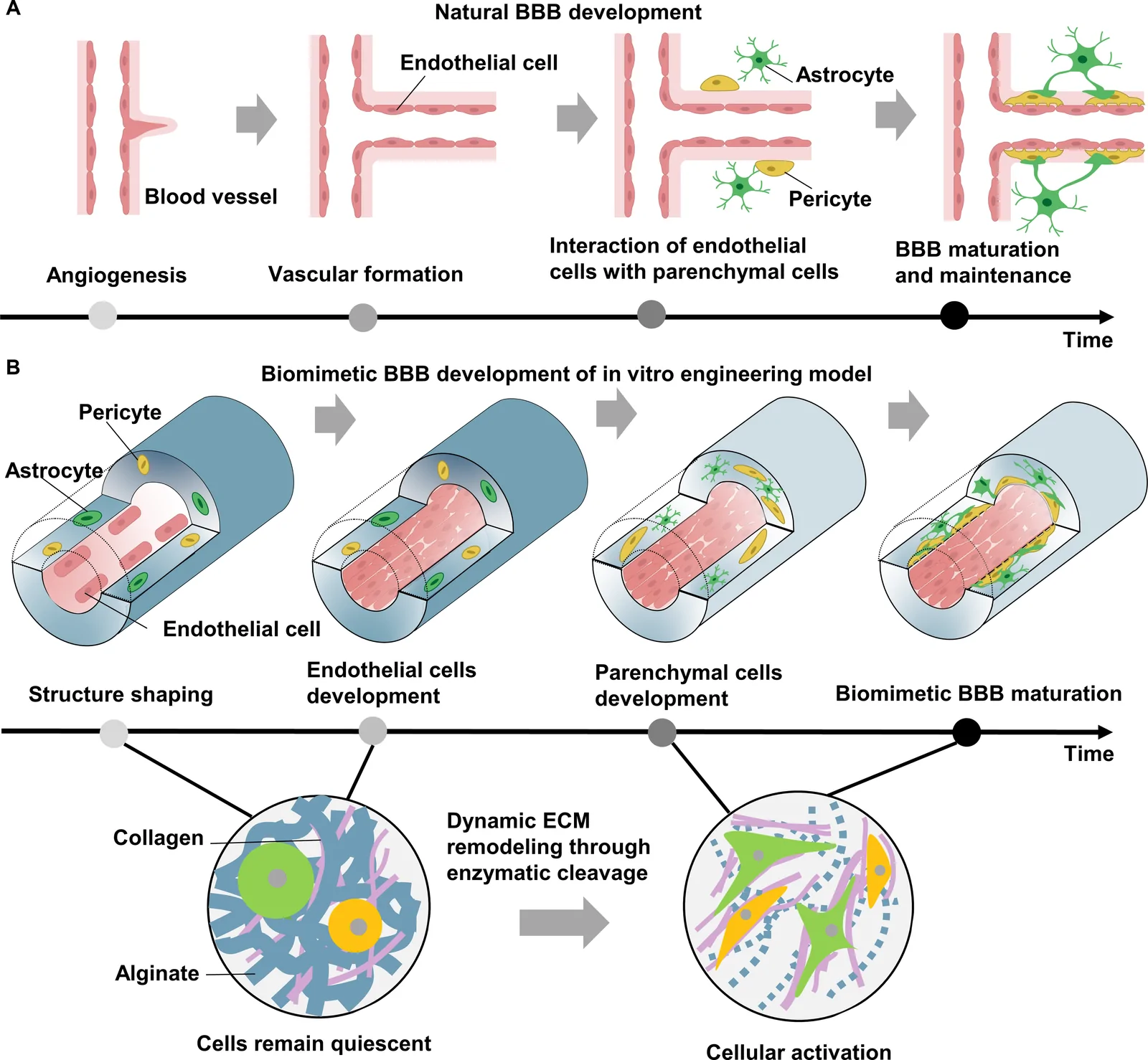
Schematic illustration of the biomimetic design concept and sequential fabrication strategy of the blood–brain barrier (BBB) model. (A) Native spatial architecture and developmental sequence of the natural BBB. The endothelial tube formation is followed by pericyte recruitment for vessel stabilization and subsequently by astrocyte integration to complete barrier function. (B) Biomimetic design and fabrication strategy of the engineered 3-dimensional (3D) BBB model with multicellular spatial and temporal characteristics. The endothelial cells first establish a functional lumen under high-stiffness matrix conditions. Enzymatic cleavage then reduces matrix stiffness and confinement, triggering sequential activation of pericytes and astrocytes and promoting barrier maturation.

To construct such type of in vitro BBB model, organ-on-chip technology—such as BBB-on-a-chip [15], miniaturized BBB [16], and synthetic microvascular systems [17]—could enable multicellular organization and controllable flow environments and improve drug screening and disease modeling [18]. However, these models typically rely on 2D cultures and therefore cannot fully represent 3D barrier structure, spatial gradients, or extracellular matrix (ECM) mechanical cues that are critical for authentic cell behaviors [19]. As a result, essential BBB features—such as endothelial organization within a cylindrical barrier geometry, spatially organized basement membrane deposition, and physiologically relevant 3D cell–cell and cell–matrix interactions—are challenging to recapitulate in vitro. To address these limitations, hydrogel-based 3D BBB models have gained increasing attention [20]. Broadly, such models follow either a bottom-up or a top-down construction strategy. The “bottom-up” approach uses stem-cell-derived organoids that self-organize under biochemical and mechanical cues, enabling developmental sequence and cell–cell communication but with limited spatial control [21]. In contrast, the “top-down” approach uses predefined scaffolds generated via casting, bioprinting, or electrospinning, followed by cell seeding to functionalize the structure [22–25]. While top-down models offer higher architectural control, they often rely on terminally differentiated cells seeded simultaneously, and the commonly used static matrix microenvironments have restrained the ability to mimic the dynamic and temporally ordered characteristics of natural tissue development.

BBB maturation in vivo is a temporally regulated process. Pericytes are recruited early to support endothelial stabilization, followed by astrocyte engagement to complete barrier function and neurovascular coupling (Fig. 1A) [26,27]. Disruption of this temporal order impairs barrier maturation and stability [28]. Concurrently, the BBB microenvironment undergoes ECM remodeling and mechanical diversification—brain tissue remains low stiffness [25] (~1 kPa), while vascular regions experience dynamic stresses from blood flow and pressure [29,30]. This mechanical heterogeneity modulates cell fate through mechanotransduction, yet current in vitro systems lack the ability to integrate such spatiotemporal mechanical dynamics.

In this study, we demonstrate that dynamic modulation of the mechanical microenvironment can serve as a mechanism for coordinating the temporally ordered activation of BBB-associated cells. The time-evolving matrix mechanics provide instructive cues that regulate transitions between quiescent and active cellular states during barrier formation. Building on this mechanobiological concept, we developed a biofabrication strategy that integrates temporally controllable modulation with 3D tissue construction to generate a spatiotemporally organized BBB (STO-BBB) model. The system adopts a hollow tubular hydrogel architecture in which endothelial cells, pericytes, and astrocytes are spatially arranged in concentric layers, enabling direct multicellular interactions without the use of artificial membranes. By decoupling initial structural assembly from subsequent cellular activation, this approach permits endothelial organization and barrier establishment to precede the engagement of supporting cells, thereby capturing key features of BBB maturation (Fig. 1B). Consequently, the STO-BBB model can be perfused, and it exhibits essential barrier properties and supports in vitro evaluation of trans-barrier transport and drug permeability within a physiologically relevant condition.

## Results

### Enzymatic cleavage remodels collagen network and facilitates rapid stress relaxation of hydrogel

To investigate how mechanical cues can regulate the sequential activation of BBB-associated cells, we first characterized the physical and viscoelastic changes of the alginate–collagen dual-network hydrogel before and after enzymatic cleavage. A series of analyses were performed, including chemical composition, collagen distribution, and rheological properties.

Fourier transform infrared (FTIR) spectroscopy analysis revealed the network transition induced by alginate–lyase treatment (Fig. 2A). Before lyase treatment, the double-network hydrogel exhibits a spectral profile closely resembling alginate [31,32], with pronounced COO– asymmetric (~1,600 cm^−1^) and symmetric (~1,415 cm^−1^) stretching bands corresponding to Ca^2+^-mediated cross-linking sites. After enzymatic cleavage, the symmetric COO– band persists, while the asymmetric band shifts toward the collagen [33] amide I region, accompanied by the clear emergence of amide II (~1,550 cm^−1^) and amide III (~1,240 cm^−1^). These features reflect selective disruption of the alginate network and increased prominence and connectivity of collagen fibrils. Collectively, the spectral shifts mark a transition from a Ca^2+^-cross-linked, alginate-dominated network to a collagen-dominated matrix after enzymatic lysing. Immunofluorescence staining revealed enhanced collagen exposure and continuity after enzymatic cleavage (Fig. 2B and C and Fig. S1). Compared with noncleaved hydrogels, which showed discontinuous collagen distribution and prominent pore-like regions, cleaved hydrogels exhibited more continuous and spatially extended collagen fibers, with increased collagen-positive area (10.87 ± 4.14% to 42.66 ± 7.10%) and mean connected component area (51.31 ± 20.66 to 251.17 ± 105.89 μm^2^). Branching-related fiber network parameters changed only modestly, suggesting that cleavage mainly enhanced overall collagen exposure and connectivity while inducing limited changes in local fibrous microarchitecture. Rheological measurements demonstrated a significant reduction in stiffness upon cleavage, with the shear modulus decreasing from 69.9 ± 17.5 Pa to 16.3 ± 6.0 Pa (Fig. 2D to F). Moreover, the stress relaxation half-time decreased markedly from 6.49 ± 1.85 s to 0.42 ± 0.05 s (Fig. 2G and H). These results indicate that enzymatic cleavage not only enhances collagen network connectivity but also reduces mechanical stiffness and accelerates stress relaxation.

**Fig. 2.**
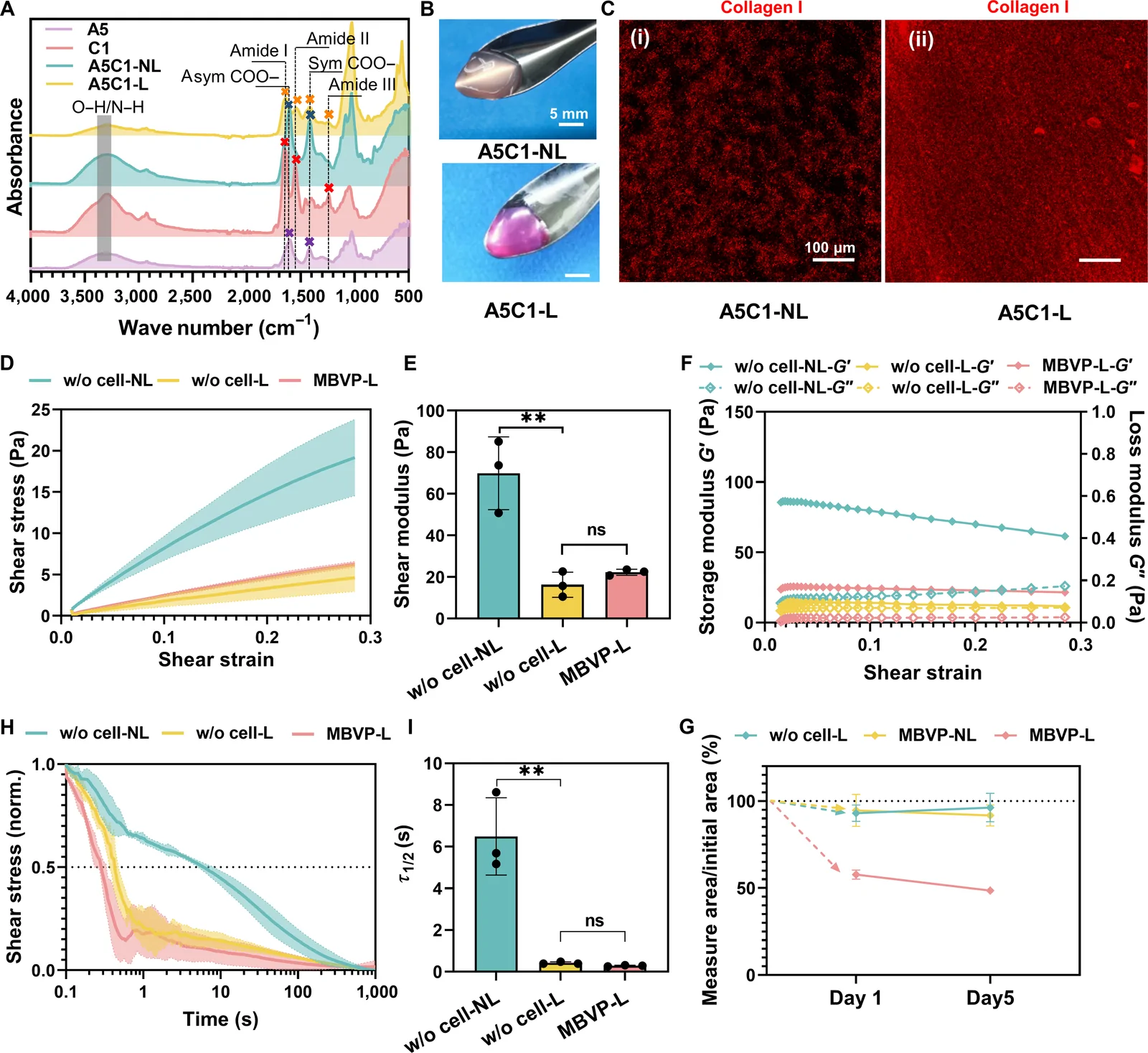
Enzymatic modulation of the properties of alginate–collagen double network hydrogels. (A) Representative Fourier transform infrared (FTIR) data of 0.5 wt% alginate (A5), 0.1 wt% collagen (C1), composite A5C1-NL (0.5 wt% alginate + 0.1 wt% collagen), and its cleaved group (A5C1-L). (B) Photograph of the A5C1-NL and A5C1-L hydrogel samples. (C) Immunofluorescence staining of type I collagen in A5C1-NL and A5C1-L hydrogels. (D to F) Shear stress–strain curves, apparent shear modulus, and storage/loss moduli of 3 hydrogel groups: acellular noncleaved A5C1 (w/o cell-NL), cleaved A5C1 (w/o cell-L), and cleaved MBVP-laden A5C1 (MBVP-L). (G and H) Stress relaxation curves and relaxation half-times of the same groups, showing significant softening and viscoelastic changes upon cleavage. (I) Macroscopic volume change of hydrogels over 5 d in culture medium, quantified by the ratio of measured to theoretical cross-sectional area. Data in (F) represent mean values; data in (D), (E), and (G) to (I) represent mean values ± SD; statistical analysis was performed using one-way ANOVA with Tukey’s multiple comparisons test, *N* = 3. **P < 0.01. ns, not significant.

### Dynamic matrix relaxation activates pericyte spreading and ECM remodeling

To assess how pericytes respond to dynamic changes in the matrix mechanics, we first compared the mechanical properties of enzymatically cleaved hydrogels with and without encapsulated cells. Although the overall shear modulus and stress relaxation properties remained comparable between the mouse brain vascular pericyte-laden, lyase-cleaved, (MBVP-L) and acellular, lyase-cleaved, (w/o cell-L) groups (Fig. 2D, E, G, and H), the presence of cells significantly altered the viscoelastic profile. The cell-laden group exhibited a higher storage modulus and lower loss modulus (Fig. 2G), indicating a shift toward a more elastic, solid-like behavior. In parallel, pericyte-laden hydrogels exhibited pronounced contraction, with cross-sectional area decreasing to 57.75 ± 2.61% on day 1 and 48.52 ± 1.09% on day 5 (Fig. 2I and Fig. S2). This mechanical shift suggests active matrix remodeling driven by pericyte–ECM interactions.

To further investigate pericyte morphological, adhesive, and matrix remodeling responses to dynamic matrix softening, we carried out immunofluorescence staining. We investigated the expression of cytoskeletal (F-actin), basement membrane (laminin), focal-adhesion (talin), and matrix components (type I collagen) markers in MBVP-L and MBVP under noncleaved (MBVP-NL) conditions at days 1 and 5. The results of costaining with F-actin/type I collagen/talin/4′,6-diamidino-2-phenylindole (DAPI) are depicted in Fig. 3A, and the results of costaining with F-actin/laminin/DAPI are shown in Fig. 3B. Larger-field representative images are shown in Fig. S3. The further quantitative analyses are shown in Fig. 3C to E. Regarding matrix remodeling, both cleaved and noncleaved groups showed signs of collagen turnover and laminin deposition, with collagen localized mainly to cell bodies and laminin surrounding the cells. In cleaved hydrogels, collagen degradation was more pronounced, and laminin secretion was enhanced and aligned with the axis of cell elongation, particularly during early stages of culture. In terms of cell adhesion and spreading, both groups exhibited focal adhesion formation colocalized with collagen, indicating integrin-mediated initial anchorage. However, in cleaved hydrogels, pericytes exhibited marked spreading and elongation, with significantly larger cell area (439.65 ± 114.87 μm^2^ versus 182.56 ± 65.70 μm^2^) and greater maximum branch length (35.09 ± 11.89 μm versus 5.39 ± 2.19 μm) than those under noncleaved conditions. Focal adhesion and actin cytoskeleton coverage were also notably larger in the cleaved group. These phenotypic changes in pericytes were further supported by transcriptomic evidence (Fig. 3F and G). RNA sequencing (RNA-seq) analysis revealed that matrix softening induced significant up-regulation of genes associated with ECM remodeling [34–40] (like FN1, COL1A1, and TNC), cell adhesion [41] (like ITGB8, TLN2, and ARHGAP24), and cytoskeletal organization [36,42–46] (like FLNB, MYL9, and MYLK).

**Fig. 3.**
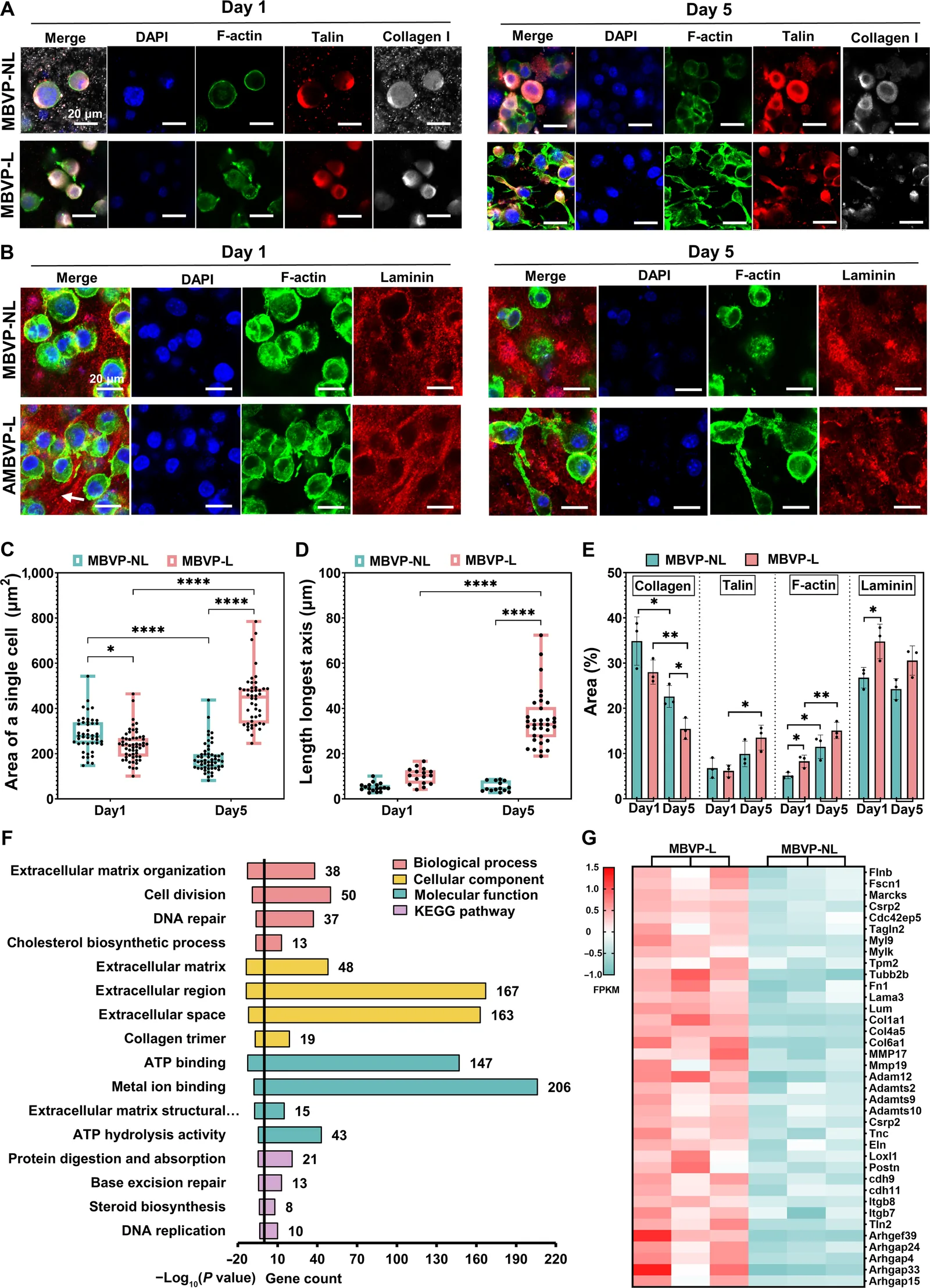
Dynamic hydrogel induced by enzymatic cleavage promotes cytoskeletal spreading and extracellular matrix remodeling of pericytes. (A) Immunofluorescence staining for type I collagen (white pseudo-color), focal adhesion protein talin (red), F-actin (green), and nuclei (4′,6-diamidino-2-phenylindole [DAPI], blue) in the mouse brain vascular pericyte (MBVP)-laden A5C1 hydrogels cultured under noncleaved (MBVP-NL) and cleaved (MBVP-L) conditions on days 1 and 5. (B) Immunofluorescence staining for F-actin (green), basement membrane protein laminin (red), and nuclei (DAPI, blue) in the MBVP-laden A5C1 hydrogels cultured under MBVP-NL and MBVP-L conditions on days 1 and 5. The white arrows in (B) indicate the possible cell migration trace in laminin matrix. (C) The average F-actin-enclosed area per cell as a measure of cell spreading. (D) Quantification of the maximum branch length per cell. Data in (C) and (D) represent mean values ± SD and were analyzed using 2-way ANOVA with Tukey’s multiple comparisons test, *N* ≥ 14. *P < 0.05 and ****P < 0.0001. (E) Quantification of the area coverage of collagen I, talin, F-actin, and laminin. Data represent mean values ± SD, statistical analysis was performed using unpaired 2-tailed Student’s *t* test, *N* = 3. *P < 0.05 and **P < 0.01. (F) GO and Kyoto Encyclopedia of Genes and Genomes (KEGG) pathway enrichment analysis based on RNA-seq of pericytes cultured in lyase-treated (MBVP-L) versus untreated hydrogels (MBVP-NL). ATP, adenosine triphosphate. (G) Heatmap of selected differentially expressed genes in MBVP-L versus MBVP-NL.

### Time-controlled matrix relaxation triggers stress-adaptive activation of astrocytes

Immunofluorescence analysis revealed that astrocytes, like pericytes, exhibited activation in response to matrix relaxation (Fig. 4A and Fig. S2C). However, unlike pericytes, this activation was not accompanied by transcriptional up-regulation of ECM or cytoskeletal genes. Instead, transcriptomic data showed prominent enrichment of Gene Ontology (GO) terms related to the endoplasmic reticulum and unfolded protein response (UPR) (Fig. 4B). Differential gene analysis revealed coordinated up-regulation of ribosomal proteins (RPL/RPS), translation initiation factors, and endoplasmic-reticulum-resident chaperones (e.g., HSPA5 and DNAJB6), indicating enhanced protein synthesis and UPR activation (Fig. 4C) [47–49]. Metabolic and antioxidant genes (TXN2, GCLC, and SOD1) were also up-regulated, reflecting redox stress buffering and glycolytic shift [50]. Mitochondrial genes (ATP5G1 and NDUFS2) showed elevated expression, suggesting enhanced oxidative phosphorylation and mitochondrial support [51]. Despite the reduced mechanical tension in the soft microenvironment, astrocytes appear to maintain metabolic activity and cytoskeletal dynamics through transcriptional up-regulation of energy-supportive pathways. Furthermore, stress-responsive transcription factors such as ATF4, CREB3L1, DDIT4, and NR4A1 were induced, forming a gene network that supports cytoprotection and adaptive remodeling [52].

**Fig. 4.**
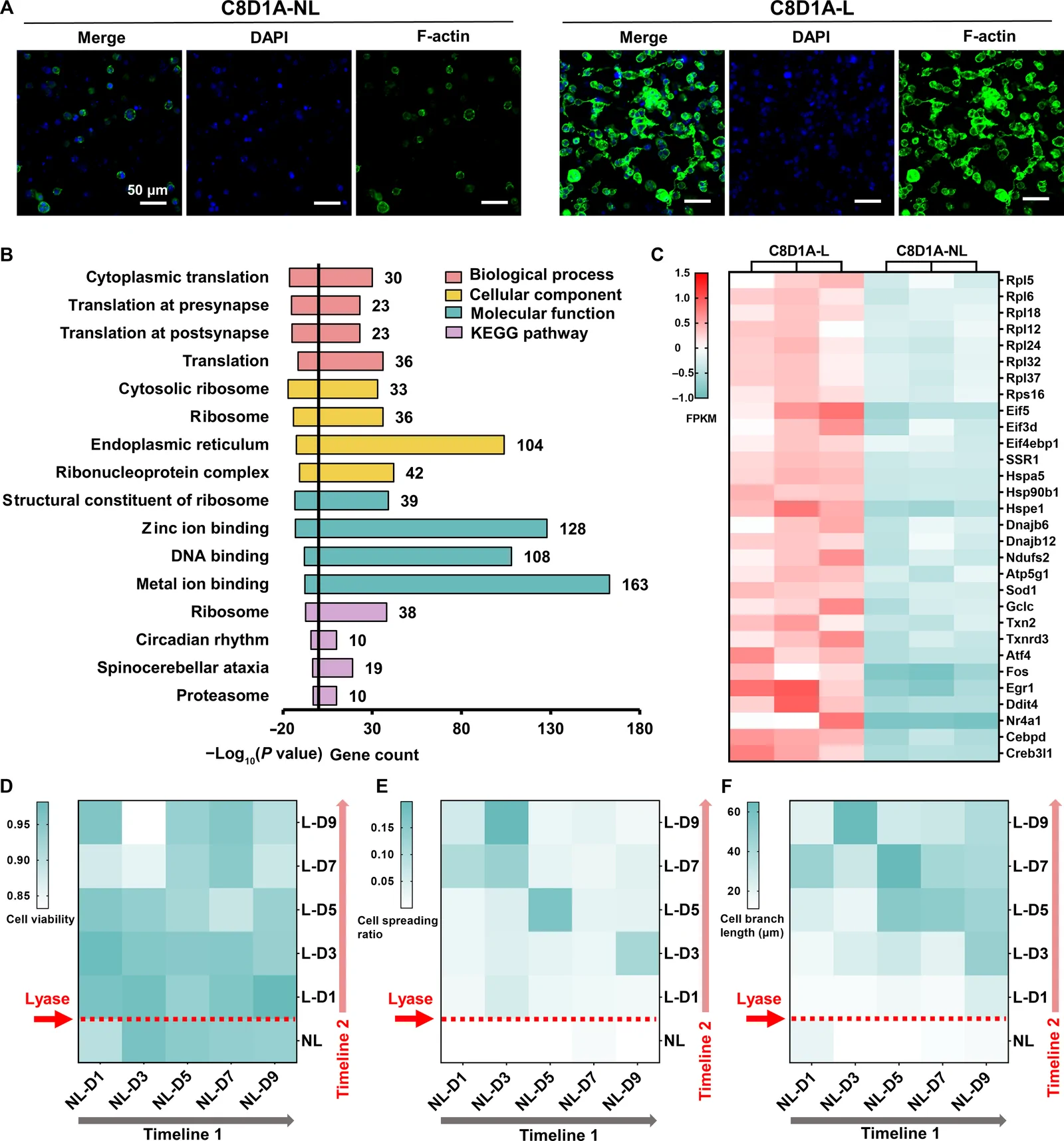
Time-dependent activation of astrocytes in dynamic hydrogels. (A) Immunofluorescence images of C8D1A-laden A5C1 hydrogels cultured under noncleaved (C8D1A-NL) and cleaved (C8D1A-L) conditions on day 5, stained for F-actin (green) and nuclei (DAPI, blue). (B) Gene Ontology (GO) and Kyoto Encyclopedia of Genes and Genomes (KEGG) pathway enrichment analysis of astrocytes under lyase-treated (C8D1A-L) versus untreated conditions (C8D1A-NL). (C) Heatmap of selected differentially expressed genes in C8D1A-L versus C8D1A-NL. (D) Cell viability across timeline 1 and timeline 2 conditions, defined as the ratio of live cells to total cells. The time-matrix experiment was designed as alginate lyase was applied at different time points during a 9-d noncleaved culture period (timeline 1), followed by an additional 9-d culture (timeline 2). Labels “NL-Dx” refer to day *x* of noncleaved culture; “L-Dy” indicates day *y* postcleavage culture (*N* = 3). (E) Cell spreading ratio across both timelines, defined as the proportion of spread cells among total viable cells (*N* = 3). (F) Quantification of average total branch length per spread cell across both timelines (*N* = 3).

To further investigate whether astrocytes remain responsive to mechanical cues at different culture stages, we implemented a time-matrix design composed of 2 sequential timelines: timeline 1 (noncleaved, continuous culture for 9 d) and timeline 2 (9-d postcleavage culture following enzymatic treatment at different time points within timeline 1). Live/dead staining results showed consistently high cell viability (>90%) throughout the 9-d period under noncleaved conditions, and only a slight decrease was observed following enzymatic cleavage at different stages (Fig. 4D). The representative staining images are shown in Fig. S4. Notably, cells under noncleaved conditions remained mostly round and nonspread, whereas cleavage at any time point led to a marked increase in spreading ratio (Fig. 4E). In addition, the average total branch length per spread cell increased progressively across the timeline 2 conditions (Fig. 4F), confirming that matrix relaxation consistently triggered astrocyte activation regardless of the cleavage time point. These findings demonstrate that astrocyte activation can be controlled along a temporal axis, using enzymatic modulation of hydrogel mechanics as an external “trigger”.

### Spatiotemporally organized construction of the STO-BBB model

Based on the above findings, we developed an STO-BBB model that integrates both structural hierarchy and staged cellular activation. As shown in Fig. 1B, the STO-BBB adopts a 3D, hollow, coaxial bilayered tubular architecture, comprising an inner confluent endothelial monolayer and an outer hydrogel layer embedding supporting cells, including pericytes and astrocytes. The model is designed to recapitulate key aspects of native BBB developmental dynamics. A multicellular BBB construct is established at the outset, followed by dynamic perfusion-driven maturation of the endothelial layer into a tubular structure, while pericytes and astrocytes remain quiescent. Subsequent hydrogel matrix cleavage and softening activate the supporting cells, which then interact with endothelial cells to promote a functional BBB maturation. The fabrication timeline is illustrated in Fig. 5A.

**Fig. 5.**
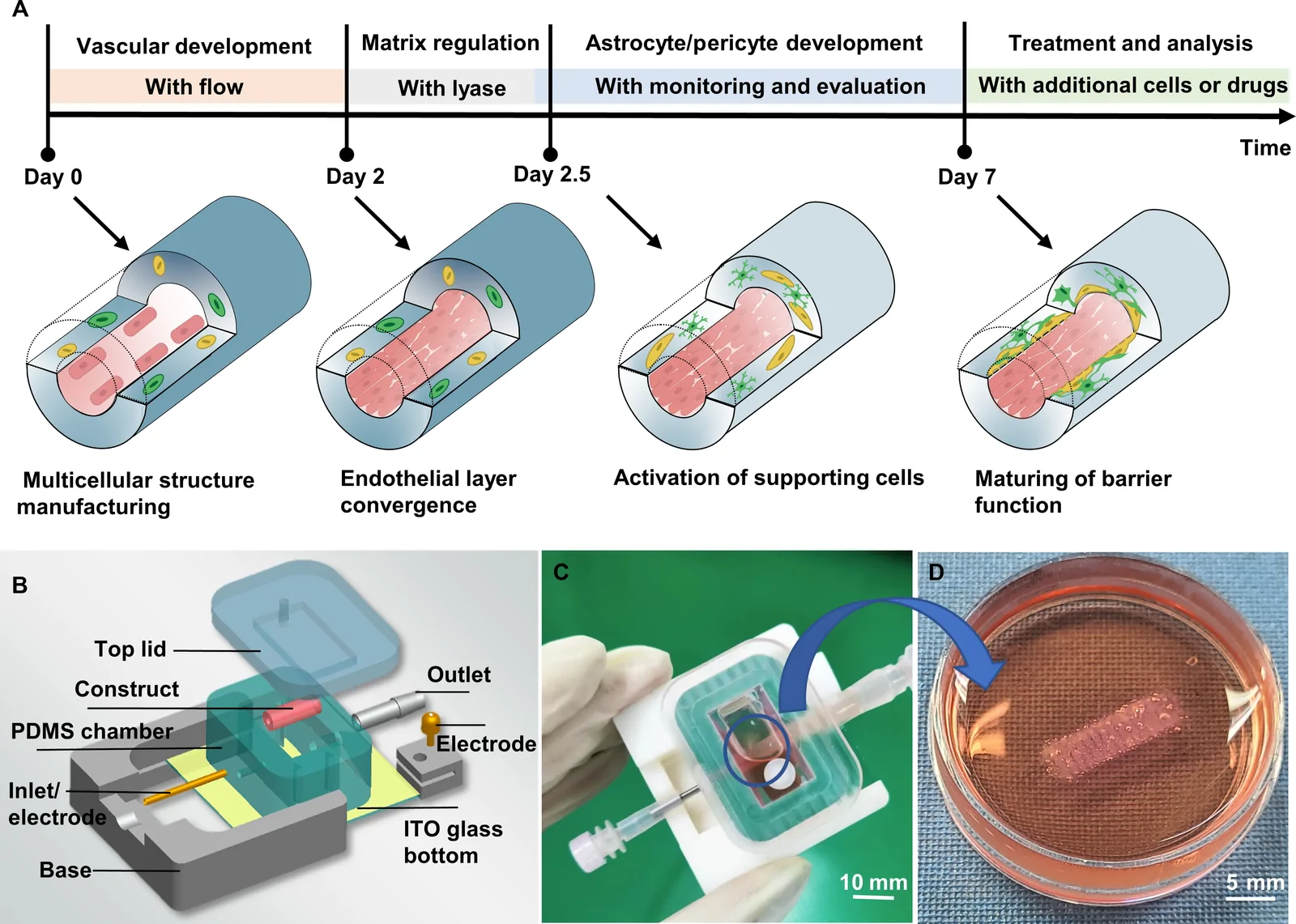
Biofabrication of the spatiotemporally organized blood–brain barrier (STO-BBB) model. (A) Experimental timeline of the biofabrication of the STO-BBB. (B) The schematic decomposition view of the BBB bioreactor. (C) Load the construct into the bioreactor. (D) Photograph of the fabricated tubular construct.

The barrier interface is established through direct multicellular contact and interaction, forming a physiologically relevant membrane without usage of any artificial membranes. This interface delineates 2 distinct compartments—the intraluminal vascular space and the extraluminal brain parenchymal space—and enables directed trans-barrier transport from the lumen to the exterior via intravascular perfusion of culture medium or test compounds. To maintain structural stability during matrix relaxation, we further developed a soft-rigid hybrid strategy that decouples macroscale structural support from microscale cellular mechanosensing (see Materials and Methods and Figs. S5A to E and S6).

To enable dynamic perfusion culture, real-time imaging, and transendothelial electrical resistance (TEER) measurements of the STO-BBB, we constructed a custom-designed bioreactor system (Fig. 5B and C). The system includes a polydimethylsiloxane (PDMS) culture chamber with inlet and outlet ports and a transparent indium tin oxide (ITO)-coated glass baseplate for optical access. The hydrogel tube is anchored within the chamber, enabling modular coculture or integration with additional tissues. For noninvasive TEER assessment, the titanium inlet and the ITO-coated baseplate [53] function as intraluminal and extraluminal electrodes, respectively. To ensure uniform endothelial coverage along the lumen [54], we used a rotatable platform: After cell suspension injection, the chamber was rotated 90° every 15 min to promote homogeneous cell attachment (Fig. S5F). The construct was subsequently perfused under dynamic flow conditions in an incubator (Fig. S5G and H). Compared with existing BBB systems [53,55–58], this platform integrates perfusion, optical monitoring, and TEER measurement within a fully 3D hydrogel architecture and accommodates sequential cellular assembly across distinct compartments.

### Formation of a confluent endothelial monolayer with intact tight junctions

To verify the establishment of a basic functional tubular endothelial barrier [59], we first fabricated a simplified construct containing only endothelial cells and evaluated it using the described method. To monitor the development of endothelial cells in real time, we transduced the endothelial cells (bEnd.3) with a lentiviral vector expressing red fluorescent protein (RFP) to enable fluorescent labeling. The macroscopic images, microscopic magnified images, and fluorescence images of the vascular constructs are shown in Fig. 6. Representative images illustrate the time-dependent development of the endothelial cell layer (Fig. 6A). Endothelial cells adhered to the substrate and formed a tightly packed, confluent monolayer after 2 d of dynamic perfusion culture. In addition, after 5 d of culture, immunofluorescence staining was performed on both the luminal surface and cross-sections to evaluate tight junction protein formation (Fig. 6B). Imaging revealed that endothelial cells uniformly lined the vascular lumen, forming a continuous 3D tubular structure (Fig. 6C and D). Localized imaging of the tube further demonstrated continuous intercellular junctions throughout the lumen (Fig. 6E). Cross-sectional images showed a well-defined and circular endothelial monolayer. High-magnification views confirmed clear expression of major tight junction proteins, including zonula occludens-1 (ZO-1), occludin, and Claudin-5 (Fig. 6F). These findings confirm successful formation of a uniform and confluent endothelial monolayer with intact tight junctions, providing a stable foundation for integrating perivascular cells and conducting downstream functional assays.

**Fig. 6.**
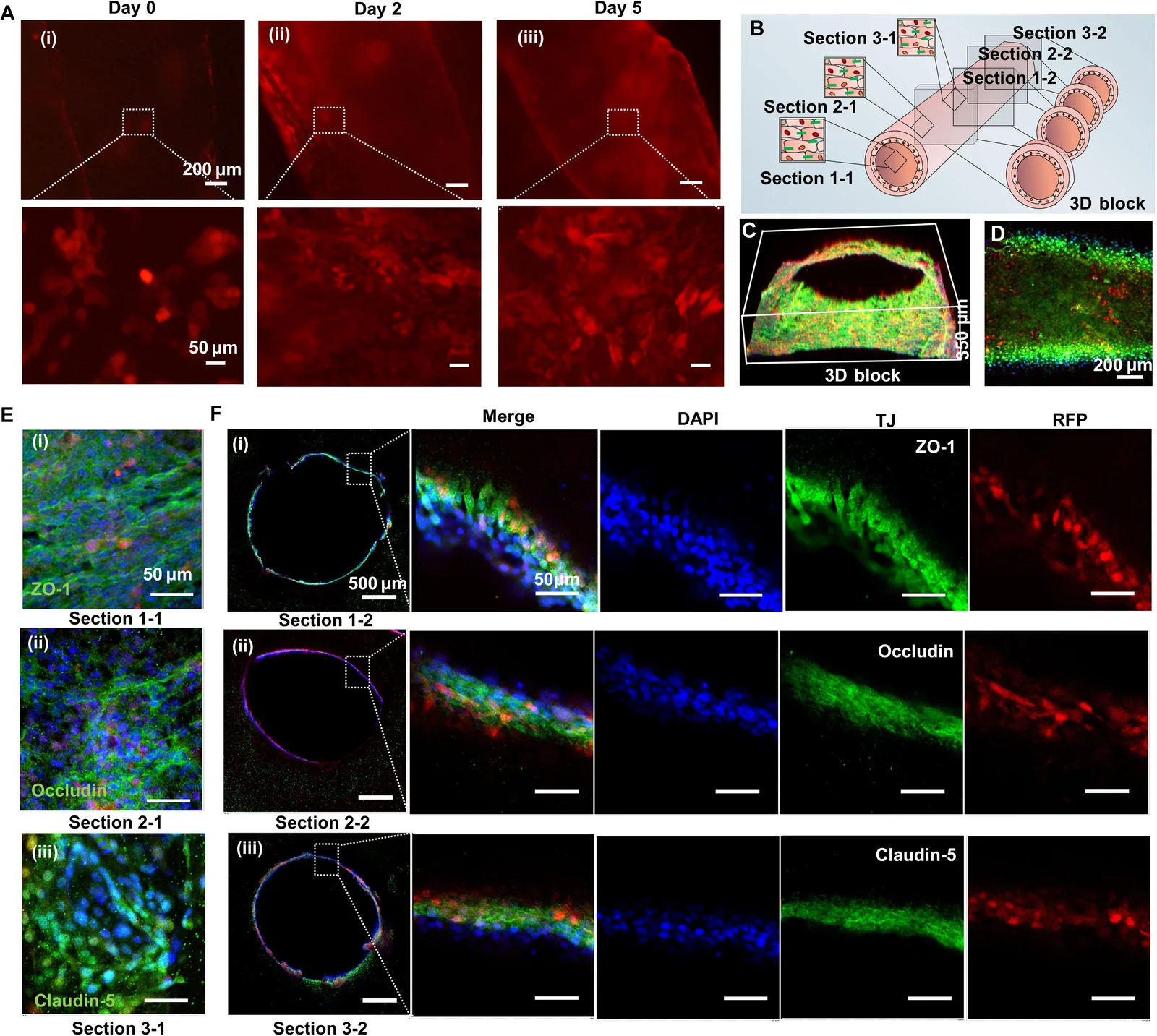
Immunofluorescence evaluation of tight junction formation in the endothelial layer. (A) Real-time observation of endothelial cells growth. (B) Schematic illustration of the staining regions. (C and D) Three-dimensional (3D) reconstruction and axial cross-section of the endothelial monolayer. Green, zonula occludens-1 (ZO-1); red: red fluorescent protein (RFP); blue, nuclei. (E) Surface immunofluorescence images of the endothelial layer. (i) to (iii) show staining for ZO-1, occludin, and Claudin-5 (green), with RFP (red) and nuclei (blue). (F) Cross-sectional view and enlarged image of the vascular wall highlighting tight junction (TJ) morphology. (i) to (iii) show staining for ZO-1, occludin, and Claudin-5 (green), with RFP (red) and nuclei (blue).

### Temporal development and multicellular organization enhance barrier function

To assess how temporal organization influences BBB formation, we compared 2 triculture models with distinct developmental sequences: a sequential triculture (Seq-Tri) group, in which endothelial cells were first allowed to form a confluent vascular structure before supporting cell activation, and a synchronous triculture (Syn-Tri) group, in which endothelial cells, pericytes, and astrocytes were cocultured from the initial time point. To verify whether supporting-cell-mediated matrix remodeling affected the endothelialized lumen, we performed cross-sectional imaging of the endothelialized tubular construct containing embedded pericytes and astrocytes at different time points. With the mechanical support of the rigid scaffold and the milder enzymatic treatment used for tubular construct preparation, the vascular structure remained largely intact, with an open lumen and RFP-positive endothelial cells distributed along the luminal boundary (Fig. S7). Fluorescent tracer permeability assays using 70-kDa fluorescein isothiocyanate (FITC)–dextran, performed on day 5 of culture, revealed markedly reduced macromolecular leakage in the Seq-Tri model relative to the Syn-Tri model, as indicated by lower extravascular fluorescence accumulation over time (Fig. 7A and B). Immunofluorescence staining conducted on day 5 showed that endothelial cells in the Seq-Tri exhibited more continuous and clearly delineated ZO-1-positive intercellular boundaries than those in the Syn-Tri (Fig. 7C), reflecting improved tight junction organization at the microscopic level. Besides, in both models, localized-vascular-sprouting-like structures, characterized by endothelial extensions projecting outward from the vessel wall, were sporadically observed in discrete regions (Fig. 7D). Quantitative analysis demonstrated a significantly lower apparent diffusion coefficient in the Seq-Tri (Seq-Tri, 1.393 × 10^−6^ ± 7.612 × 10^−7^ cm/s; Syn-Tri, 3.487 × 10^−6^ ± 2.796 × 10^−7^ cm/s; Fig. 7E and F). In parallel, TEER measurements showed that the Seq-Tri model maintained higher resistance values throughout culture and reached a significantly greater peak TEER at day 9 compared with the Syn-Tri model (Seq-Tri, 112.677 ± 8.245 Ω·cm^2^; Syn-Tri, 62.062 ± 6.134 Ω·cm^2^; Fig. 7G and H), indicating enhanced overall barrier function.

**Fig. 7.**
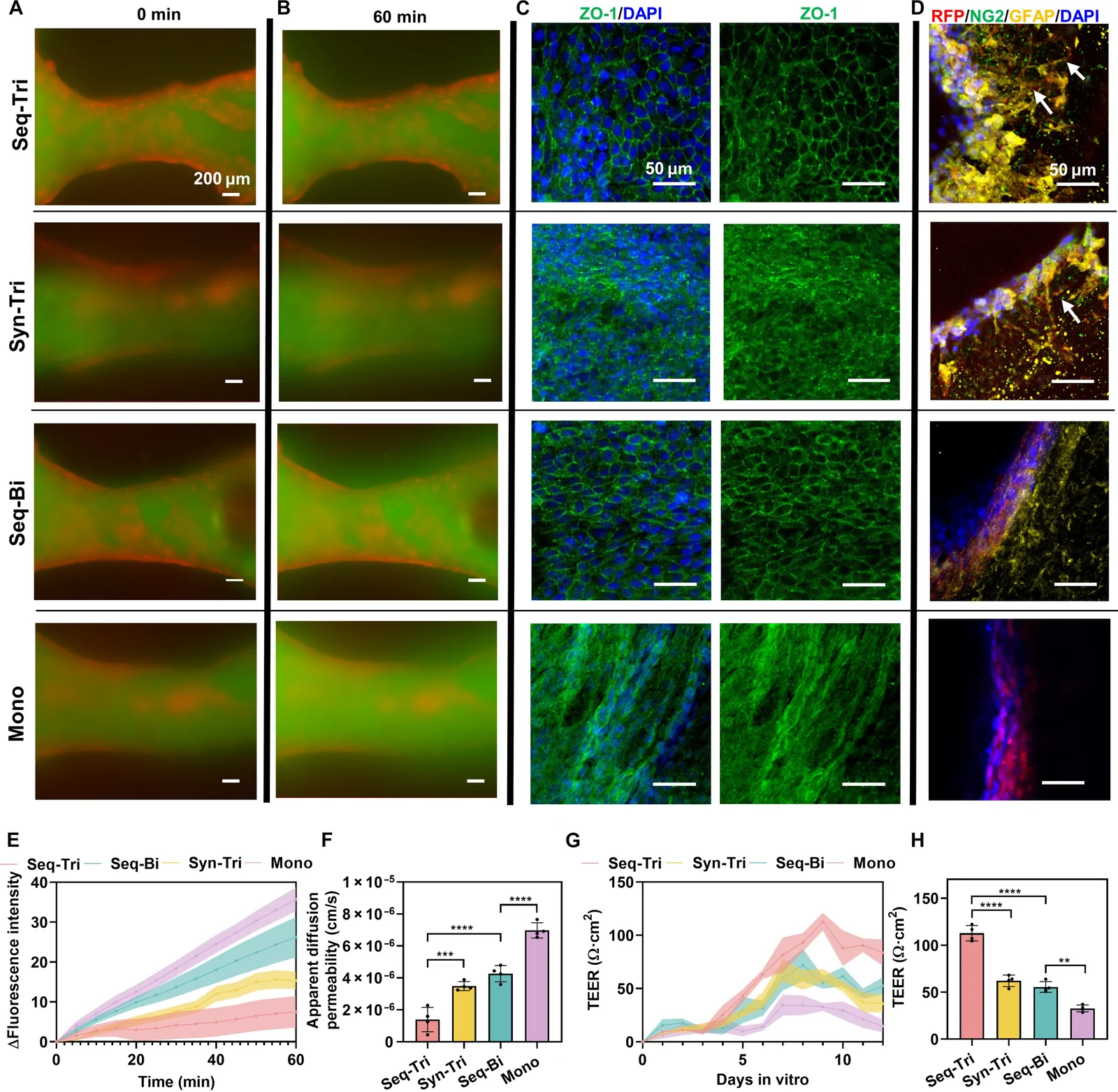
Functional evaluation of the multicellular blood–brain barrier (BBB) model. Grouping for the in vitro BBB model: Seq-Tri indicates the sequential triculture group of endothelial cells, pericytes, and astrocytes; Syn-Tri indicates the synchronous triculture group of endothelial cells, pericytes, and astrocytes; Seq-Bi indicates the sequential biculture group of endothelial and astrocytes; and Mono indicates the monolayer culture group of endothelial cells. (A and B) Fluorescence microscopy images taken at 0 and 60 min after perfusion of 70-kDa FITC–dextran on day 5 among 4 groups. (C) Immunofluorescence images of the tight junction proteins zonula occludens-1 (ZO-1) on day 5 among 4 groups. (D) Immunofluorescence images of vessel wall regions on day 5 among 4 groups. White arrows indicate vascular sprouting. (E) Dynamics of normalized external fluorescence intensity over time for the 4 groups. Fluorescence intensity was quantified as the mean gray value of the green channel in the external region of the tubes. Fluorescence intensity was baseline-corrected by setting the first time point (*t* = 0) to zero, and subsequent values were calculated as the increase relative to this baseline (the graph shows the mean values ± SD in the form of a shaded envelope of the normalized fluorescence intensity, *n* = 4). (F) Quantification of the apparent diffusion permeability in each group. (G) Transendothelial electrical resistance (TEER) values were measured using the integrated bioreactor every day. TEER was baseline-corrected by setting the first time point (day 0 before involving endothelia cells) to zero, and subsequent values were calculated as the increase relative to this baseline in every group (the graph shows the mean values ± SD in the form of a shaded envelope of the TEER values at 1 kHz, *n* = 4). (H) Comparison of TEER values among 4 groups on day 9. Data represent mean values ± SD; statistical analysis was performed using one-way ANOVA with Tukey’s multiple comparisons test. **P < 0.01, ***P < 0.001, and ****P < 0.0001.

To further dissect the contribution of supporting cell types to BBB formation under sequential culture conditions, we compared the triculture model (Seq-Tri) with an endothelial–astrocyte biculture model (Seq-Bi) and an endothelial monoculture model (Mono), which contains only endothelial cells. Immunofluorescence analysis revealed that astrocyte could improve endothelial tight junction organization, as evidenced by sharper and more clearly defined ZO-1-positive intercellular boundaries in the Seq-Bi compared with Mono (Fig. 7C). The triculture displayed the most pronounced junctional definition. Besides, localized-vascular-sprouting-like features were predominantly observed in pericyte-containing cultures and were absent under the Seq-Bi and Mono conditions (Fig. 7D). Barrier permeability exhibited a graded dependence on cellular complexity, with the Seq-Tri model showing the lowest permeability (1.393 × 10^−6^ ± 7.612 × 10^−7^ cm/s), followed by the Seq-Bi (4.266 × 10^−6^ ± 5.086 × 10^−7^) and Mono (6.975 × 10^−6^ ± 4.771 × 10^−7^) models (Fig. 7E and F). TEER measurements showed a corresponding trend, with resistance values on day 9 progressively decreasing from Seq-Tri to Seq-Bi and Mono (Seq-Tri, 112.677 ± 8.245 Ω·cm^2^; Seq-Bi, 55.533 ± 5.758 Ω·cm^2^; Mono, 32.708 ± 3.633 Ω·cm^2^; Fig. 7G and H), indicating that the incorporation of multiple supporting cell types enhances overall barrier performance. Besides, the phase angle and capacitance were measured at the same time among 4 groups; the results (Fig. S8A and B) showed that the phase angle and capacitance gradually increase with the culture time and reach a peak on the fifth day, followed by some fluctuations among groups, which may indicate the process of cell proliferation.

### A BBB–glioma composite model enables evaluation of drug-selective permeability

To assess the functional relevance of the engineered STO-BBB model for pharmacological applications, we established a composite in vitro system by embedding a glioma-containing hydrogel layer around the BBB tube. The resulting coaxial construct reproduced the spatial relationship between tumor tissue and the vascular barrier (Fig. 8A and B). A glioma-only construct without BBB components was fabricated as a control.

**Fig. 8.**
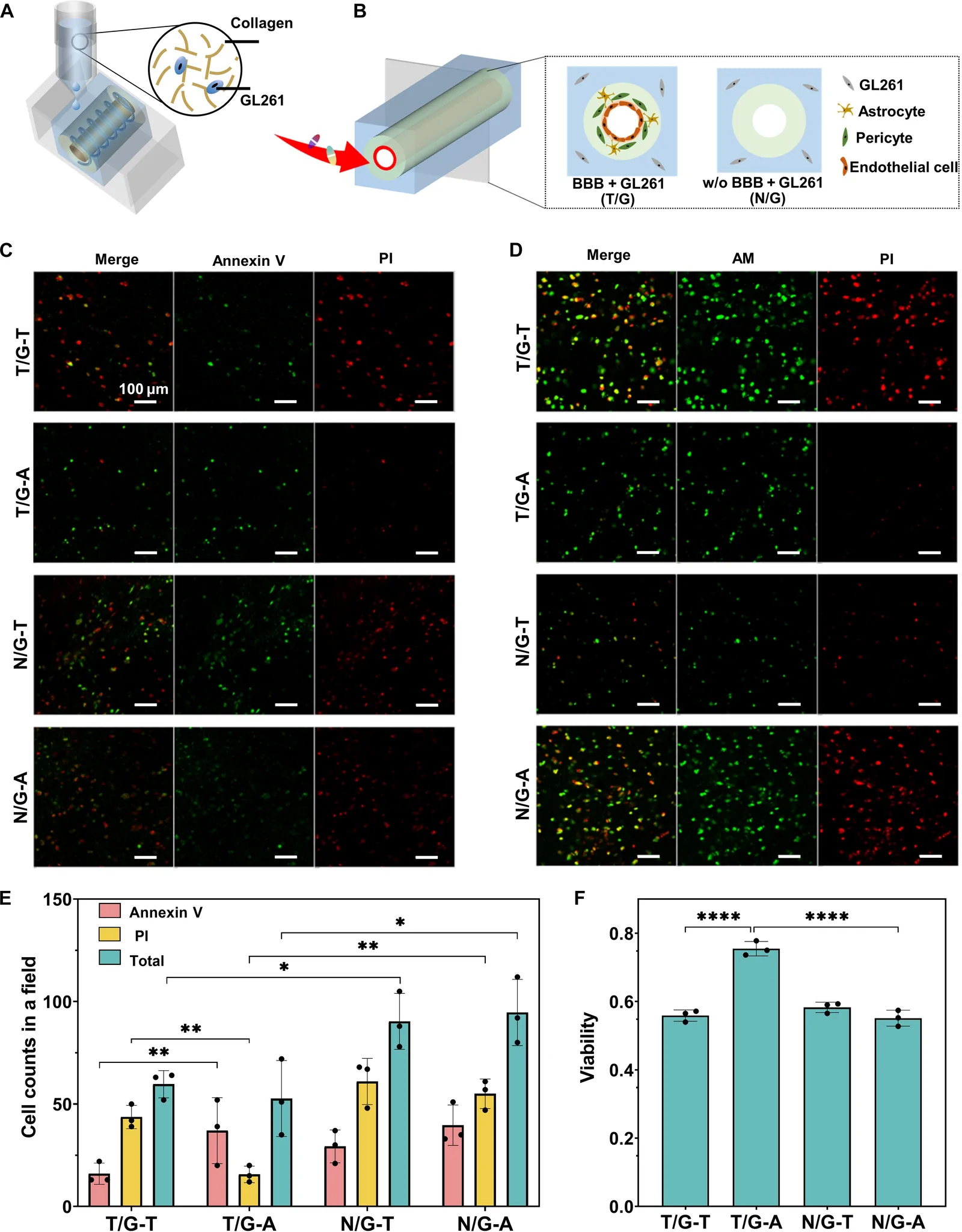
Construction of the composite blood–brain barrier (BBB) glioma model and evaluation of drug effects on glioma. (A) Schematic diagram of the construction method for the composite BBB glioma model, where collagen hydrogel containing glioma cells is poured in situ outside the already developed BBB, for the experimental group; a BBB model without cells but with other conditions the same as the experimental group is used as the control. (B) Schematic diagram of drug permeability experiments for the BBB glioma model (experimental group BBB + GL261 [T/G]) and the non-BBB glioma model (control group w/o BBB + GL261 [N/G]). (C and D) Apoptosis and live/dead staining of glioma cells in different groups. T/G-T represents the presence of the BBB with temozolomide treatment; T/G-A represents the presence of the BBB with doxorubicin treatment; N/G-T represents the absence of the BBB with temozolomide treatment; N/G-A represents the absence of the BBB with doxorubicin treatment. (E) Quantification of apoptotic cells in different groups. Data represent mean values ± SD; statistical analysis was performed using unpaired 2-tailed Student’s *t* test, *N* = 3. *P < 0.05 and **P < 0.01. (F) Viability of glioma cells in different groups. Data represent mean values ± SD; statistical analysis was performed using one-way ANOVA with Tukey’s multiple comparisons test, *N* = 3, ****P < 0.0001.

Two types of chemotherapeutic drugs with distinct BBB permeability profiles, i.e., doxorubicin (DOX; nonpenetrating) and temozolomide (TMZ; BBB-permeable) were tested. Drug administration was performed via vascular perfusion, and glioma responses were evaluated using apoptosis staining and live/dead assays (Fig. 8C and D). Quantification of apoptotic cell counts and cell viability is shown in Fig. 8E and F.

TMZ induced similar levels of apoptosis and viability reduction in both the BBB–glioma and glioma-only models, indicating efficient penetration through the engineered barrier. In contrast, DOX exhibited significantly reduced cytotoxicity in the composite model, with glioma cells showing higher viability and lower apoptosis compared to the glioma-only condition. Within the BBB–glioma model itself, TMZ consistently produced stronger therapeutic effects than DOX.

## Discussion

Temporal ordering is a critical feature of natural tissue development and functioning. However, most biofabrication approaches used for the development of 3D in vitro tissue models rely on direct replication of tissue structure, without accounting for the temporal sequence required for the growth and maturation of distinct cell types. Consequently, such types of approaches are inherently static and oversimplified. In this study, we reframe the fabrication process from a dynamic perspective, not only reconstructing complex cellular components and biomimetic tissue architecture but also recapitulating dynamic tissue developmental processes. Accordingly, we develop a biofabrication method in which cell growth is induced by dynamic microenvironmental changes, enabling on-demand activation of specific cell types along the developmental timeline of engineered tissue models. Using this approach, we successfully construct a biomimetic 3D in vitro BBB model exhibiting temporally ordered development.

The dynamic microenvironmental regulation described above is achieved through the enzymatic cleavage of the alginate–collagen double-network hydrogel matrix: Before enzymatic treatment, cells encapsulated within the matrix remain in a quiescent state for several days; after cleavage, the cells can be rapidly activated. By exploiting this property, the in vitro BBB tissue model can be guided through a sequential developmental process after initial structure shaping. We first promoted the endothelialization of the hydrogel lumen, while supporting cells, including pericytes and astrocytes, are maintained in a quiescent state. Supporting cells are subsequently activated after establishment of a continuous endothelial lining, thereby achieving an ordered, on-demand multicellular maturation process.

The enzymatic matrix modulation experiments focused on pericytes and astrocytes because the dynamic hydrogel microenvironment was designed to regulate the abluminal supporting cell compartment rather than to directly stimulate endothelial cells. The compatibility of endothelial cells with this alginate/collagen-based matrix system, including cell viability, proliferation, and tight junction protein expression after enzymatic remodeling, has been systematically demonstrated in our previous study [60]. Therefore, in the present work, endothelial responses were evaluated in the complete 3D BBB model. By analyzing matrix changes following enzymatic cleavage together with the response patterns of pericytes and astrocytes, we elucidate the mechanisms by which dynamic microenvironmental transitions trigger cellular state changes. Prior to enzymatic cleavage, alginate constitutes the dominant network within the matrix, partitioning collagen fibers into discontinuous microstructures and conferring high matrix stiffness with slow stress relaxation. Under this highly mechanically constrained microenvironment, cells maintain high viability and are able to adhere to collagen, yet remain in a nonspreading, quiescent state. Following enzymatic cleavage of the matrix, removal of alginate allows collagen to gradually become the dominant structural network. This transition markedly enhances collagen network connectivity while substantially reducing mechanical constraint. The resulting more compliant microenvironment rapidly drives pericytes toward an activated phenotype, characterized by increased proliferation, migration, and cell spreading, as well as active ECM remodeling, including collagen degradation and laminin deposition. These collective changes further give rise to pronounced macroscopic tissue contraction. Previous studies have investigated the influence of matrix stiffness on cell fate. For example, high-stiffness alginate hydrogels can induce cellular dormancy [61], whereas low-stiffness alginate matrices favor cell proliferation and sustained activity [62]. However, most existing approaches provide only a static mechanical microenvironment that is either stiff or compliant and remains unchanged throughout the entire culture period. In contrast, the strategy presented here enables controlled transitions of the matrix microenvironment, thereby allowing regulation of cellular states in a manner that is aligned with distinct developmental stages of the in vitro BBB model. Beyond stiffness, stress relaxation is also a critical regulator of cell behavior. Prior work has shown that rapidly relaxing hydrogels [63] (*τ* ≈ 70 s) significantly promote cell spreading and proliferation, effects closely associated with integrin-mediated adhesion, cytoskeletal tension regulation, and focal adhesion assembly. Notably, the hydrogel used in this study exhibits an exceptionally rapid stress relaxation behavior following enzymatic cleavage (*τ* ≈ 1 s), comparable to that of natural brain tissue and substantially faster than most previously reported materials [43,64]. This ultrafast stress relaxation enables cells to respond rapidly upon external triggering: constructs contracted to 57.75 ± 2.61% of their initial size within 1 d, followed by a slower decrease to 48.52 ± 1.09% by day 5. A recent review [65] summarized 2 primary mechanisms by which dynamically regulated matrices influence cell behavior: (a) integrin-mediated adhesion and (b) reduction of mechanical constraint accompanied by enhanced capacity for tension dissipation. Our dynamic hydrogel strategy integrates both mechanisms. During the initial stage, collagen-derived adhesive ligands support integrin signaling, while the highly mechanically constrained matrix does not permit cell spreading. Following enzymatic cleavage, the reduction in mechanical constraint enables pericytes to rapidly remodel their surrounding matrix microenvironment, accompanied by pronounced cell spreading and migration. A schematic illustration of these mechanisms is depicted in Fig. 9. Notably, the enhanced ECM activity observed in pericytes within the BBB model is consistent with the physiological functions of pericytes in vivo [26–28]. For instance, in vivo, pericytes express high basal levels of FN1 particularly in perivascular regions such as the brain, where they contribute to basement membrane maintenance and vascular stabilization [34,35]. Similarly, pericytes cultured on enzymatically lysed matrix in this study exhibited significantly increased FN1 expression.

**Fig. 9.**
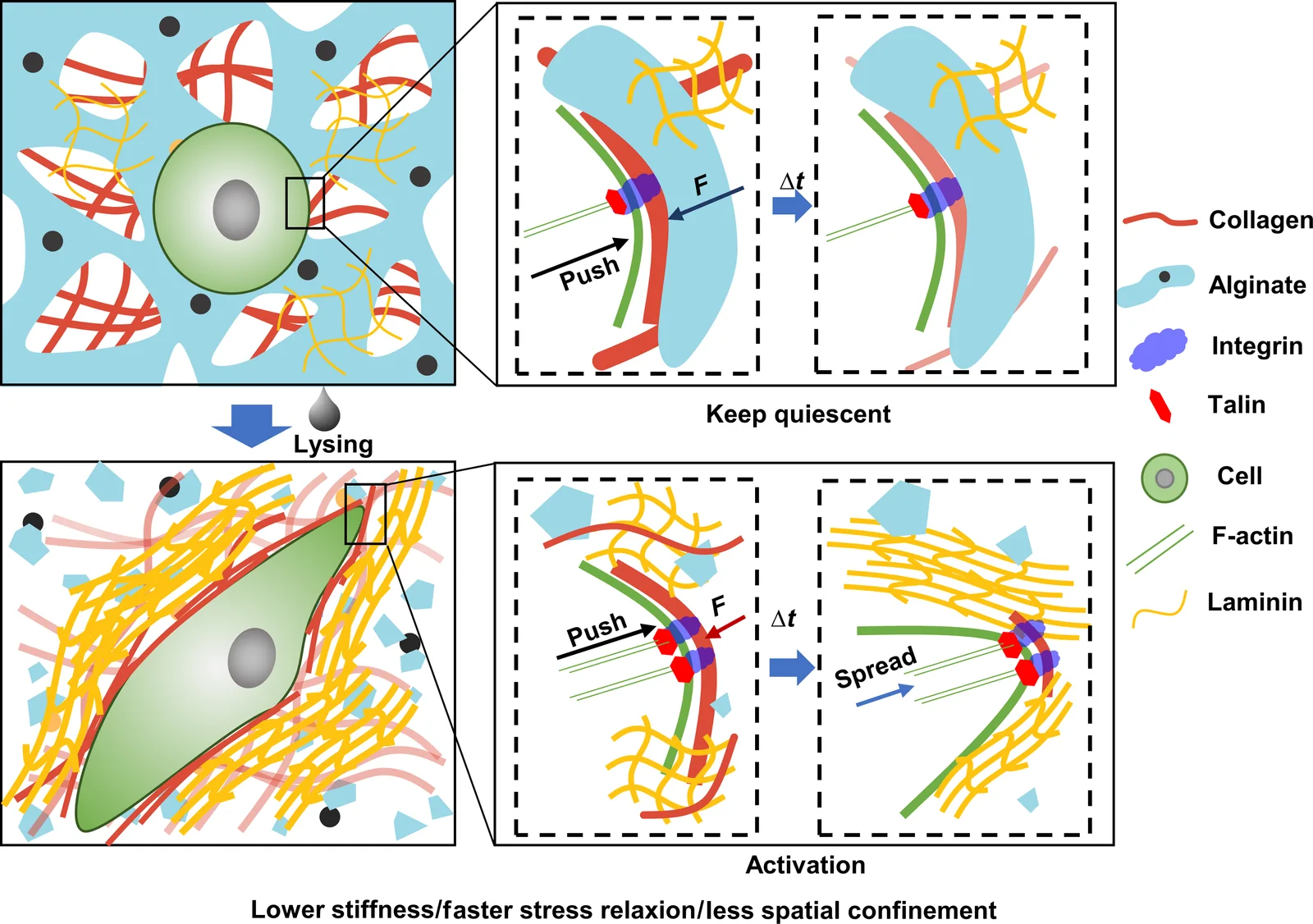
Schematic illustration of the proposed mechanism by which hydrogel lysing modulation regulates pericyte behavior.

Astrocytes exhibited a response pattern to microenvironmental changes that was clearly distinct from that of pericytes. Transcriptomic analysis revealed that astrocytes activated adaptive stress and metabolic regulatory programs, including the UPR, mitochondrial support pathways, and redox-buffering mechanisms. Previous studies have shown that astrocytes display limited spreading in stiff matrices with slow stress relaxation [66]. However, their transcriptional responses to microenvironments with rapid stress relaxation have not been systematically investigated. Our results indicated that astrocytes may interpret reduced matrix mechanical constraint as a signal to enhance biosynthetic capacity and metabolic activity, a response consistent with their roles in maintaining tissue homeostasis and supporting neuronal function in the CNS [26–28]. Together, these findings demonstrate that pericytes and astrocytes exhibit distinct responses to dynamic microenvironmental changes and that the functional activation of each cell type closely aligns with its physiological role in the native BBB. This may arise from the low-constraint matrix environment, which permits different cell types to self-organize according to their intrinsic behavioral programs. Such a property is particularly critical for the construction of highly biomimetic multicellular tissue models.

Based on these findings, we constructed a BBB model (STO-BBB) that integrates biomimetic multicellular composition, structural features, and temporally ordered development. Our results demonstrated that cellular composition and developmental sequence significantly influence the functional performance of the 3D in vitro BBB models. Compared with synchronously cocultured controls, the sequentially developed models exhibited lower macromolecular leakage and higher TEER values, indicating that stepwise and ordered cellular development is more favorable for barrier maturation. It should be noted that *R*_blank_ of TEER calculating was measured on day 0 before endothelial cell seeding and used as a sample-specific baseline correction to reduce initial variations among devices, hydrogel constructs, and electrode contacts. However, during long-term culture, hydrogel remodeling, cell-mediated matrix contraction, medium exchange, and ionic fluctuations may alter the electrical background of the system, which could affect the absolute TEER values. Therefore, the TEER values reported here should be interpreted mainly as apparent values for comparative analysis between groups rather than absolute endothelial-specific resistance values. The functional enhancement observed under sequential developmental conditions may arise from several factors. Allowing endothelial cells to reach a certain level of maturation before activation of supporting cells may reduce competition for nutrients and physical space, thereby facilitating the establishment of tight junctions. In addition, synchronous coculture may expose endothelial cells to disordered paracrine cues, whereas temporally ordered culture separates cellular signaling at different developmental stages along the time axis, thereby strengthening productive cell–cell interactions. Most existing in vitro BBB models either culture multiple cell types simultaneously or culture supporting cells prior to endothelial seeding [22,24,53,55,67]. In contrast, the model presented here more closely reflects the physiological sequence of multicellular development observed in natural barrier tissue. Together, these results suggest that temporal ordering represents a critical design parameter for optimization of in vitro BBB models.

Consistent with previous reports, multicellular composition further enhanced barrier integrity [26,55,68–70]. The astrocytes improved the distribution of tight junction proteins, and coculture of astrocytes and pericytes with endothelial cells enhanced overall barrier function. In pericyte-containing cultures, localized-vessel-sprouting-like structures were observed but not reducing overall barrier properties like permeability and TEER. It may result from spatial heterogeneity in matrix remodeling, collagen exposure, supporting cell activation, and local cell distribution after enzymatic modulation, which may create permissive local regions for endothelial extension into the surrounding matrix. This is consistent with the role of pericytes in regulating endothelial sprouting during vascular remodeling and supporting BBB integrity during maturation [69,71]. Compared with previously reported BBB models based on murine cell lines [72,73], the STO-BBB model developed in this study exhibits improved barrier function under temporally ordered culture conditions, with an approximately onefold increase in TEER. Furthermore, the functional validity of the STO-BBB model was demonstrated in a BBB–glioma coculture system. Drug permeability assays using TMZ and DOX successfully recapitulated clinically observed differences in BBB permeability, confirming the physiological relevance of the engineered barrier model [74–76]. Owing to its modular design and compatibility with perfusion-based drug delivery systems, this model is well suited for applications including evaluation of CNS drug transport, screening of nanocarriers, assessment of immunotherapeutic strategies, and investigation of tumor–vascular interactions.

Nevertheless, several limitations should be acknowledged. First, the use of murine-derived cell lines may limit the translational predictive accuracy of the model for human BBB physiology. Future studies should validate the key findings using human-derived primary BBB-related cells or human-cell-based BBB models. Barrier integrity and drug permeability-selective profiles should be further confirmed in human-derived models to improve the translational relevance of this platform. In addition, natural BBB development involves complex and tightly regulated spatiotemporal signaling cascades [26,28], which cannot be fully recapitulated by staged cell introduction alone. Thus, the sequential activation strategy used here should be viewed as an engineered approximation rather than a complete reconstruction of natural BBB development. Furthermore, incorporation of additional cellular components, such as neurons and immune-related cells, represents an important direction for constructing more functional BBB models and may further expand the applicability of this system [6]. Moreover, although dynamic matrix regulation enables controlled cellular activation, more refined control over submicron-scale ECM architecture or biochemical permeability gradients could further improve developmental fidelity. Future efforts should focus on achieving region-specific and finely tunable modulation of matrix properties, in combination with activation of multiple cell types at distinct time points, to enable biofabrication of tissue and organ models with more biomimetic spatiotemporal organization.

## Conclusion

This study proposes and validates a strategy of broad applicability in which dynamic regulation of the matrix microenvironment enables temporally ordered control over multicellular tissue development. The resulting STO-BBB model exhibits functional properties that more closely approximate physiological conditions, providing an evolving biomimetic 3D in vitro BBB model for investigating neurovascular development, modeling disease processes, and evaluating CNS-targeted therapeutic strategies. From a more general perspective, biofabrication approaches that integrate advanced technologies such as 3D bioprinting with matrix-triggered regulation of cellular states offer a new paradigm for in vitro construction of complex living tissue models of superior physiological relevance. In this paradigm, tissue function is no longer determined solely by initial structural configuration but instead emerges from the coordinated development of multiple cell types under precisely controlled temporal sequences. Such an approach may help bridge the critical gap between conventional in vitro models based on direct tissue replication and natural tissues and organs that undergo dynamic, ordered development.

## Materials and Methods

### Experimental design

We designed the sodium alginate/collagen double-network hydrogel and conducted comparative analyses of its FTIR spectra, microstructure, mechanical properties, and cellular responses before and after treatment with alginate lyase. Then, we explored the mechanisms of pericytes and astrocytes activation derived by microenvironmental changes. Subsequently, we constructed a biomimetic BBB model incorporating endothelial cells, pericytes, and astrocytes in a coaxial tubular structure, along with a chip system capable of perfusion and real-time monitoring. By using time-controlled microenvironment regulation, stepwise activation of multiple cell types within the BBB model was conducted. We then comparatively analyzed the effects of cellular composition and temporal factors on BBB function. Finally, glioma cells were introduced to the abluminal side of the BBB model, and the inhibitory effects of different drugs after crossing the barrier were evaluated to assess the model’s selective permeability.

### Cell culture

Mouse brain microvascular endothelial cells (bEnd.3) and glioma cells (GL261) were obtained from the Cell Bank of the Chinese Academy of Sciences (China). MBVPs were purchased from Bluef (Shanghai) Biotechnology (China), and type I astrocytes (C8D1A) were obtained from iCell Bioscience (China). bEnd.3 cells were transduced with an RFP-expressing lentiviral vector by iCell Bioscience. The resulting red fluorescent cells were referred to as bEnd.3-RFP. All cell types were cultured in complete medium composed of 89% (v/v) high-glucose Dulbecco’s modified Eagle’s medium (SH30022.01, HyClone), 10% (v/v) fetal bovine serum (SA201.02, CellMax), and 1% (v/v) penicillin–streptomycin (SV30010, HyClone) and maintained in a humidified incubator at 37 °C with 5% CO_2_. Cells were subcultured or harvested when they reached 80% to 90% confluence. Only cells with passage numbers below 10 were used for all experiments.

### Preparation of low-stiffness hydrogels

#### Acellular hydrogel preparation

Soft dual-component hydrogels composed of sodium alginate (180947, Sigma-Aldrich) and collagen (custom-extracted from rat tail [60]) were prepared using a 2-step cross-linking approach. A precursor solution containing 0.5 wt% sodium alginate and 0.1 wt% collagen was mixed on ice and adjusted to pH 7.6 using 0.5 mol·l^−1^ NaOH. The solution was cast into PDMS (SYLGARD 184, Dow Corning) molds (10 mm × 10 mm × 2 mm) and incubated at 37 °C for 20 min to allow thermally induced self-assembly of the collagen network. The gels were then carefully transferred to 1 wt% CaCl_2_ solution and incubated for an additional 20 min to achieve ionic cross-linking of alginate. Hydrogels were designated as A5C1 when referring to material composition, and as w/o cell-NL when used as acellular controls in comparison with cell-laden groups. To obtain lyase-treated hydrogels, the formed constructs were soaked overnight in complete culture medium containing alginate lyase (0.2 μg/ml; A1603, Sigma-Aldrich) at 37 °C. Subsequently, samples were rinsed 3 times with lyase-free complete medium at 1-h intervals. The lysed hydrogels were designated as A5C1-L or w/o cell-L. Control hydrogels composed of either 0.5 wt% sodium alginate alone (A5) or 0.1 wt% collagen alone (C1) were prepared using their own gelling method. All procedures were performed under sterile conditions.

#### Cell-laden hydrogel preparation

MBVP and C8D1A cells were harvested at ~80% confluence, resuspended in the alginate–collagen precursor solution (0.5 wt% alginate and 0.1 wt% collagen) at a final density of 4 × 10^6^ cells/ml. Gelation and enzymatic treatment with alginate lyase were performed following the same procedures as for acellular hydrogels. Experimental groups were defined as MBVP-NL and MBVP-L and C8D1A-NL and C8D1A-L, respectively.

### Characterization of low stiffness hydrogel properties

#### Chemical composition

FTIR spectroscopy (Nicolet iS10, Thermo Fisher Scientific) was used to analyze the chemical composition of hydrogel constructs and to assess the retention and removal of alginate and collagen components. Samples from groups A5C1, A5C1-L, A5, and C1 were freeze-dried for 48 h and analyzed as intact dried hydrogel discs without further grinding. Spectra were collected over the range of 4,000 to 400 cm^−1^ with a resolution of 4 cm^−1^ and 32 scans per sample.

#### Viscoelasticity

Viscoelasticity of the hydrogels was evaluated using a rotational rheometer (MCR302, Anton Paar). Samples included acellular gels (w/o cell-NL and w/o cell-L) and cell-laden gels (MBVP-L) that had been cultured for 5 d prior to testing. Measurements included stress relaxation, shear stress–strain response, and strain-dependent storage (*G*′) and loss (*G*″) moduli. All tests were conducted in hydrated conditions using a 25-mm parallel plate geometry (gap, 1 mm) at 22 °C. Prior to testing, samples were equilibrated in phosphate-buffered saline (PBS; HyClone) for 30 min.

#### Strain sweep

Shear stress, storage (*G*′), and loss (*G*″) moduli were recorded under 1-Hz frequency as strain increased from 1% to 30%, providing insight into nonlinear viscoelastic behavior and mechanical stability under deformation. For shear stress–strain analysis, linear fitting was performed on the initial linear region of each curve, and the resulting slope was defined as the apparent shear modulus. This value was used to compare the shear stiffness among experimental groups.

#### Stress relaxation

A constant strain of 15% was applied, and the stress decay was recorded over 1,000 s to characterize the time-dependent viscoelastic behavior of the hydrogels. For comparison across groups, stress relaxation data were normalized to a scale of 0 to 1, with the initial peak stress set to 1. The relaxation half-time, defined as the time required for the normalized stress to decay to 0.5, was used as a representative parameter to quantify viscoelastic relaxation behavior.

#### Macroscopic contraction

To assess hydrogel volume contraction potentially induced by pericyte activity, the surface area of hydrogels from groups w/o cell-NL, w/o cell-L, and MBVP-L was measured on days 1 and 5 of incubation. Samples were imaged and planar surface area was quantified using ImageJ. To estimate relative volume change, measured surface areas were compared to the theoretical design area (10 mm × 10 mm = 100 mm^2^).

### Live/dead staining

To dynamically evaluate C8D1A cell behavior before and after matrix cleavage, a dual-timeline strategy was implemented:

• Timeline 1: Cells were cultured under noncleaved conditions for 9 consecutive days.
• Timeline 2: On days 1, 3, 5, 7, or 9 of timeline 1, alginate lyase was applied to enzymatically cleave the alginate component of the hydrogel. After cleavage, cells were cultured for an additional 9 d.

Live/dead staining was performed on the dual-timeline strategy. At each time point, 3 parameters were assessed: cell viability, spreading ratio, and total protrusion length. Samples were incubated with a calcein acetoxymethyl ester (calcein-AM)/propidium iodide (PI) staining solution (KGAF001, KeyGEN BioTECH) at 37 °C for 30 min in the dark, following the manufacturer’s instructions. Fluorescence images were acquired using a confocal laser scanning microscope (A1, Nikon). Viable (green) and dead (red) cells were quantified using ImageJ. Cell viability was calculated as the ratio of live cells to total cells. Besides, cell morphology was analyzed based on calcein-AM fluorescence images to evaluate spreading behavior. A cell was defined as “spread” if it exhibited at least one visible branch. The cell spreading ratio was calculated as the number of spread cells divided by the total number of live cells in each field of view. For each spread cell, the total branch length was obtained by summing the lengths of all visible branches, measured using the line tool in ImageJ.

In addition, this live/dead staining protocol was also applied to evaluate glioma cell viability in the subsequent BBB–glioma coculture model, as described in later sections.

### Immunofluorescence staining

At defined time points, hydrogel samples were fixed with 4% paraformaldehyde (BL539A, Biosharp) for 4 h, washed 3 times with PBS, and then incubated overnight at 4 °C in a PBS-based working solution containing 0.3% Triton X-100 (T6200G, Biotopped), 5% bovine serum albumin (AR0009, Boster), and the appropriate primary antibodies. This step was performed to simultaneously achieve permeabilization, blocking, and primary antibody binding. On the following day, samples were washed 3 times with PBS and incubated with fluorescently labeled secondary antibodies for 1 h at room temperature in the dark. Nuclei were counterstained with DAPI (5 μg/ml; T19827, TargetMol). After staining, all samples were mounted and imaged using a confocal laser scanning microscope.

Staining targets and antibody combinations were as follows:

• Acellular hydrogels (w/o cell-NL and w/o cell-L): Fixed and stained to assess collagen network structure before and after cleavage. Primary antibody was mouse anti-type-I collagen (66761-1-Ig, Proteintech; 1:200), and secondary antibody was goat anti-mouse immunoglobulin G (IgG) 647 (ab150115, Abcam; 1:400). Collagen fluorescence images were quantitatively analyzed using ImageJ under identical processing parameters for all groups. The collagen-positive area ratio was calculated as the percentage of collagen-positive pixels relative to the total image area after consistent threshold segmentation. Connected collagen-positive regions were identified using 8-neighbor connectivity, and the mean connected component area was calculated to evaluate the continuity of collagen-positive regions. To further characterize the local fibrous network, the binary collagen images were converted into fiber centerlines, from which the mean branch length and branch-point density were quantified. Branch-point density was normalized to the collagen-positive area and expressed as junctions per square millimeter of collagen-positive area.
• Pericyte-laden hydrogels (MBVP-NL and MBVP-L): Samples were collected on days 1 and 5 of culture. Two multiplex staining combinations were applied:

1. Focal adhesion and cytoskeleton remodeling group: Primary antibodies are mouse anti-type-I collagen (66761-1-Ig, Proteintech; 1:200) and rabbit anti-talin 1 polyclonal antibody (14168-1-AP, Proteintech; 1:100); additional markers are CoraLite Plus 488-Phalloidin (PF00001, Invitrogen; 1:200) and DAPI. Secondary antibodies are goat anti-mouse IgG 647 and monkey anti-rabbit IgG 594 (A-21207, Invitrogen; 1:200).
2. ECM remodeling group: Primary antibody is rabbit anti-laminin (ab11575, Abcam;1:200); additional markers are CoraLite Plus 488-Phalloidin and DAPI. Secondary antibody is monkey anti-rabbit IgG 594.

Image analysis was performed using ImageJ to quantify fluorescence area ratios per marker, average cell area per field, and the maximum branch length of cells in the region of interest.

In addition, astrocyte-laden hydrogels (C8D1A-NL and C8D1A-L) on day 5 of culture were stained using the same multiplex immunostaining protocol as used for pericytes to evaluate astrocyte adhesion and cytoskeletal remodeling.

For the subsequent BBB model, key functional proteins were also assessed via immunofluorescence staining, with detailed results presented in later sections.

### RNA-seq and analysis

RNA-seq was performed with 3 biological replicates per group. Total RNA was extracted using RNAiso Plus (Takara, Japan). RNA concentration and quality were assessed using an ND-2000 spectrophotometer (NanoDrop Technologies) and an Agilent 5300 Bioanalyzer. Samples with an OD260/280 (optical density at 260 and 280 nm) ratio of 1.8 to 2.2, OD260/230 ≥ 2.0, RNA quality number ≥ 6.5, 28*S*/18*S* ≥ 1.0, and a total RNA amount > 1 μg were used for library preparation. RNA purification, reverse transcription, library preparation, and sequencing were performed by Shanghai Majorbio Bio-Pharm Biotechnology Co. Ltd. (Shanghai, China) according to the manufacturer’s instructions (Illumina, San Diego, CA, USA). Stranded mRNA libraries were prepared from 1 μg of total RNA using the Illumina Stranded mRNA Prep Ligation kit. Polyadenylated RNA was enriched, fragmented, and reverse-transcribed into cDNA. The libraries were sequenced on an Illumina NovaSeq X Plus platform to generate 2 × 150-bp paired-end reads. Raw reads were filtered using fastp [77] and aligned to the reference genome using HISAT2 [78]. The mapped reads were assembled using StringTie [79], and gene expression was quantified as fragments per kilobase of transcript per million mapped reads (FPKM). Differences between the experimental and control groups were assessed using an unpaired 2-tailed *t* test. Genes with a mean expression fold change ≥ 1.5 and *P* < 0.05 were considered up-regulated and subjected to GO and Kyoto Encyclopedia of Genes and Genomes (KEGG) enrichment analyses using DAVID. Terms and pathways with an adjusted *P* value of <0.05 were considered significantly enriched.

### High-stiffness bioink optimization

To develop printable hydrogels capable of structurally supporting the BBB model, various hydrogel formulations based on sodium alginate (alginate), gelatin methacryloyl (GelMA; GM90, EFL), and hyaluronic acid methacrylate (HAMA; 150K, EFL) were screened. Candidate formulations included A50G50 (5 wt% alginate + 5 wt% GelMA), A50G100 (5 wt% alginate + 10 wt% GelMA), A50G50H25 (5 wt% alginate + 5 wt% GelMA + 2.5 wt% HAMA), G50H50 (5 wt% GelMA + 5 wt% HAMA), G100H50 (10 wt% GelMA + 5 wt% HAMA), and A25G50H50 (2.5 wt% alginate + 5 wt% GelMA + 5 wt% HAMA).

GelMA and HAMA hydrogels were prepared by dissolving the corresponding lyophilized polymer foams in 0.25% (w/v) lithium phenyl-2,4,6-trimethylbenzoylphosphinate (EFL) solution under 37 °C water bath conditions and protected from light to obtain hydrogel precursor solutions. The obtained precursor solutions were then mixed according to the designed concentrations to prepare the composite hydrogel precursor solution. The mixture was injected into PDMS molds (10 mm × 10 mm × 2 mm), followed by ionic cross-linking with 1 wt% CaCl_2_ for 20 min at 22 °C and subsequent photocrosslinking under 405-nm light for 2 min. Formed hydrogels were carefully removed for rheological analysis.

Rheological tests were conducted using a rotational rheometer. Shear storage modulus (*G*′) was used as a measure of stiffness. The time-dependent shear viscosity curves of each formulation were recorded and fitted to calculate the slope, serving as a printing stability index. For the A50G50 formulation, cyclic strain testing (0.01% ↔ 5%, 3 cycles) was performed to evaluate shear thinning and structural recovery behavior based on *G*′ and *G*″ responses.

Based on mechanical stiffness, printability, and viscosity stability, the A50G50 formulation was selected as the structural support material for subsequent BBB model constructing. The stability of the A50G50 supportive scaffold after enzymatic treatment was further evaluated using rigid, soft, and hybrid cuboid samples with identical external dimensions of 8 mm × 8 mm × 6 mm. In the hybrid samples, a helical A50G50 scaffold was embedded within the soft A5C1 matrix. After treatment with alginate lyase (0.2 μg/ml) at 37 °C overnight, the rigid A50G50 material showed no significant decrease in compressive modulus, while the hybrid samples retained a significantly higher modulus than the soft matrix alone.

### Design and fabrication of the custom perfusable bioreactor system

To enable the sequential fabrication, coculture, and functional assessment of a BBB model, a custom-designed multifunctional perfusable bioreactor platform was developed. The system comprises 3 functional modules: the molding/culture chamber, perfusion interface, and electrode interface, all mounted on a 3D-printed photopolymer resin base to ensure structural alignment and stability.

The molding/culture chamber was fabricated from PDMS using a computer numerical control (CNC)-machined poly(methyl methacrylate) mold. The PDMS chamber measured 15 mm × 27 mm × 17.5 mm with an 8-mm wall thickness. Aligned through-holes were formed on the front and rear walls: 1.85 mm (inlet) and 5.6 mm (outlet) in diameter, centered at 6 mm above the base, for connection to perfusion ports. The chamber was sealed at the top with a CNC-machined polycarbonate lid containing a vent port compatible with a 0.22-μm sterile air filter.

The perfusion interface consisted of a titanium inlet tube (outer diameter of 2 mm and wall thickness of 0.3 mm) and a CNC-machined polycarbonate outlet connector (inner diameter of 5.6 mm and wall thickness of 0.3 mm). Both connectors were press-fitted into the corresponding preformed holes in the PDMS chamber and connected to medical-grade silicone tubing. A peristaltic pump (RZ1030B, Runze Fluid) delivered adjustable flow for continuous perfusion.

The electrode interface enabled noninvasive, real-time monitoring of TEER. The PDMS chamber was bonded to an ITO-coated glass slide (size, 50 mm × 50 mm × 1 mm; coating thickness, ~220 nm; resistance, ~6 Ω/sq; transmittance, ~84%; Guluo Glass). The ITO surface was immersed in 5% (v/v) 3-aminopropyltriethoxysilane (ST1087, Beyotime) aqueous solution for 30 min at 60 °C, thoroughly rinsed with ultrapure water, and dried at 60 °C. The PDMS surface was then treated with oxygen plasma (oxygen flow, 2 min; power, 40%; treatment time, 30 s; Diener electronic Zepto) and immediately brought into contact with the treated ITO surface to form a stable bond. A gold-plated compression spring contact was attached to the ITO surface. In the assembled system, the titanium inlet tube served as the intravascular electrode, while the ITO-coated glass acted as the extravascular electrode.

The assembled system is autoclavable, compatible with standard incubator conditions, and suitable for 3D tissue modeling, perfusion-based stimulation, and functional evaluation experiments.

### Sequential reconstruction of the BBB model

The BBB model developed in this study consisted of a hollow tubular hydrogel structure with defined geometry (inner diameter, 2 mm; outer diameter, 5.6 mm; length, 20 mm). The model incorporated 3 cell types with spatially distinct organization: bEnd.3 cells formed a confluent monolayer on the inner lumen surface, while MBVP pericytes and C8D1A astrocytes were embedded within the hydrogel wall, forming a 3D layer.

The hydrogel tube adopted a soft/rigid hybrid design, wherein a spring-shaped rigid scaffold was embedded within a soft, cell-laden hydrogel matrix. The rigid scaffold was composed of 5 wt% alginate and 5 wt% GelMA (A50G50), providing mechanical support, while the soft matrix consisted of 0.5 wt% alginate and 0.1 wt% collagen (A5C1), serving as a cell-permissive ECM mimic.

The fabrication process involved 3 sequential steps:

• Step 1: Fabrication of the soft/rigid hybrid hydrogel tube. The spring-shaped rigid scaffold (A50G50 hydrogel) was fabricated via embedded bioprinting into a support bath composed of 4.5 wt% gelatin and 1 wt% CaCl_2_, prepared following previously reported methods. Printing was conducted at 22 °C using a 23-gauge nozzle, with an extrusion pressure of 300 mbar and a speed of 4 mm/s. The scaffold had an inner diameter of 3.8mm, a pitch of 1 mm, and a filament diameter of 0.5 mm. After printing, the scaffold was placed into a custom-designed mold, followed by casting of a soft hydrogel precursor containing MBVP and C8D1A cells, each at a density of 2 × 10^6^ cells/ml (total, 4 × 10^6^ cells/ml). After gelation of the collagen and ionic cross-linking of the alginate, the soft/rigid hydrogel construct was carefully demolded.
• Step 2: Endothelial cell seeding and perfusion culture. The hydrogel tube was transferred into the bioreactor and connected to inlet and outlet channels. Both ends were sealed with low-melting-point agarose to ensure fluid isolation. A bEnd.3 cell suspension (1 × 10^7^ cells/ml) was injected into the lumen, and the device was mounted on a rotary platform rotating 90° every 15 min for 2 h to ensure uniform cell attachment along the inner wall. Perfusion was then initiated at a flow rate of 0.2 ml/min and continued for 48 h to promote endothelial layer formation and functional maturation.
• Step 3: Enzymatic stiffness modulation and multicellular activation. After perfusion, the construct was treated with complete medium containing alginate lyase (0.05 μg/ml) at 37 °C for 4 h to reduce the matrix stiffness. The medium was then replaced with fresh, lyase-free complete medium, which was subsequently exchanged 3 times at 1-h intervals. The mechanical modulation activated both pericytes and astrocytes, triggering a transition from a quiescent to an active state. These responses contributed to the structural and functional maturation of the in vitro BBB model.

Group definitions for functional comparison were as follows:

Seq-Tri: The sequential triculture group of endothelial cells, pericytes, and astrocytes. In this group, the experimental set was as described above.

• Syn-Tri: The synchronous triculture group of endothelial cells, pericytes, and astrocytes. In this group, the pericytes and astrocytes were activated at day 0 after endothelial cells seeding.
• Seq-Bi: The sequential biculture group of endothelial and astrocytes. In this group, the hydrogel tube contains only astrocytes.
• Mono: The monolayer culture group of endothelial cells. In this group, the system contains only endothelial cells.

### Functional characterization of the reconstructed BBB

#### Permeability test

The endothelial barrier function was evaluated using 70-kDa FITC–dextran (46945, Sigma-Aldrich) at a final concentration of 10 μM in endothelial cell growth medium. The tracer was perfused through the vascular lumen, and then the constructs were incubated under static conditions in an incubator at 37 °C for 1 h to allow molecular diffusion. Fluorescence images were captured using an inverted fluorescent microscope (Ti-S, Nikon), and intensity was quantified using ImageJ. The apparent diffusion permeability coefficient (*P*_app_) was calculated on the basis of the following equation:

$$P_{\mathrm{app}}=\left[1/\left(I_{1}-I_{b}\right)\right]\times \left(dI/dt\right)\times \left(d/4\right)$$ (1)

where *I*_1_ is the initial fluorescence intensity, *I*_b_ is the background fluorescence intensity, d*I*/d*t* is the change rate of the fluorescence intensity over time, and *d* is the diameter of the tube.

#### Key protein expression analysis related to BBB

After 5 d of culture, samples from group Mono were fixed and immunostained to assess the junctional integrity of the endothelial monolayer. Primary antibodies included rabbit anti-ZO-1 (21773-1-AP, Proteintech), rabbit anti-occludin (27260-1-AP, Proteintech), and rabbit anti-Claudin 5 (34-1600, Invitrogen), each used at a 1:200 dilution. Secondary antibody was goat anti-rabbit IgG 488 (ab150077, Abcam; 1:200). For comparison of tight junction formation among different culture groups, ZO-1 immunostaining was also performed in the Mono, Seq-Bi, Syn-Tri, and Seq-Tri groups. To further characterize cellular composition and organization across groups, immunofluorescence staining was performed for pericyte and astrocyte markers. Neuron-glial antigen 2 was used to identify pericytes, with a rabbit anti-neuron-glial antigen 2 primary antibody (YN2308, Immunoway; 1:200) and goat anti-rabbit IgG 488 secondary antibody. Astrocytes were stained for glial fibrillary acidic protein using rat anti-glial-fibrillary-acidic-protein primary antibody (13-0300, Invitrogen; 1:200) and goat anti-rat IgG 555 secondary antibody (ab150158, Abcam; 1:200). Endothelial cells were visualized on the basis of their intrinsic red fluorescence. Confocal imaging was used to observe multicellular spatial organization and protein expression patterns within the hydrogel structure. Staining followed the protocol described above.

#### TEER measurement

To evaluate the dynamic development of endothelial barrier integrity, TEER was measured daily starting from the day of endothelial cell seeding until day 12. An AC signal of 1-kHz frequency and 200-mV amplitude was applied using a high-precision inductance, capacitance, and resistance meter (LCR-6000, GW Instek, China).

The raw resistance values were converted to TEER (Ω·cm^2^) using the following equation:

$$\text{TEER}=\left(R_{\text{sample}}-R_{\text{blank}}\;\right)\times A$$ (2)

where *R*_sample_ is the resistance of the endothelial construct, *R*_blank_ is the resistance of the system on day 0 before involving endothelia cells, and *A* is the luminal surface area (cm^2^).

Besides, the phase angle and capacitance were measured at the same time among 4 groups.

### Construction of the BBB–glioma model and drug evaluation

To assess drug permeability, a BBB–glioma model (T/G) was constructed by embedding GL261 glioma cells (4 × 10^6^ cells/ml) in 0.2 wt% type I collagen around the hydrogel-based vascular construct, forming a coaxial structure with a total cross-section of 9 mm × 9 mm. A placeholder was used to define the tumor region and removed after gelation. The BBB structure (T group) was precultured with 2 d of endothelial perfusion, followed by enzymatic hydrogel softening and 5 additional days of culture for barrier maturation. Glioma cells (G) were added on day 7.

As a control, a non-BBB glioma model (N/G) was constructed using the same hydrogel tube geometry but without any BBB-associated cells (endothelial, pericytes, and astrocytes). In both models, the outer chamber was filled with collagen containing glioma cells.

After 2 d of coculture, both models were perfused with TMZ (5 μM) or DOX (5 μM), followed by 24 h of static incubation (the experimental groups were defined as Table 1). After that, apoptosis staining (commercial kit) and live/dead assays were performed in the tumor region. Confocal images were analyzed using ImageJ to quantify viability and apoptosis.

**Table 1.** Grouping information for drug evaluation experiments

Model configuration	Drug	Abbreviation
BBB + GL261	Temozolomide	T/G-T
	Doxorubicin	T/G-A
GL261	Temozolomide	N/G-T
	Doxorubicin	N/G-A

### Statistics and reproducibility

All quantitative data are presented as means ± SD from at least 3 independent samples. Statistical analyses were performed using GraphPad Prism version 8.3.0 (GraphPad Software, San Diego, CA, USA). Two-tailed Student’s *t* test was applied for comparisons between 2 groups. One-way analysis of variance (ANOVA) was used for single-factor comparisons, and 2-way ANOVA for dual-factor experiments, followed by Tukey’s multiple comparisons test. Differences were considered statistically significant at *P* < 0.05.

## Data Availability

The data that support the findings of this study are available from the corresponding author upon reasonable request.
