# Supplementary material for: Microenvironment-Driven 3-Dimensional Biofabrication Enables Sequential Maturation of the Blood–Brain Barrier
Source: Research (Wash D C). 2026 Oct 5;9:1408. doi: 10.34133/research.1408 (PMC13636311; doi:10.34133/research.1408)
Supplement: Supplementary file 1 [file research.1408.f1.zip › Revised Supplementary Data_clean_FV.docx]

**Fig. S1. Quantitative analysis of collagen fiber network remodeling after enzymatic cleavage.** (A) Collagen-positive area ratio, indicating the overall exposure of collagen fibers within the hydrogel matrix. (B) Mean connected component area, reflecting the continuity of collagen-positive regions. (C) Mean branch length and (D) branch-point density, describing local fiber-network morphology and branching characteristics. Enzymatic cleavage markedly increased collagen-positive area and connected component area, while branching-related fiber-network parameters showed relatively modest changes. Data are presented as mean ± SD, n = 3. Statistical significance was determined by unpaired two-tailed Student’s t-test. *p < 0.05, **p < 0.01.

**Fig. S2**. Macroscopic volume change of hydrogels on day 1 and day 5 in culture medium, quantified by the ratio of measured to theoretical cross-sectional area and visualized with overlays on the designed 10 mm × 10 mm outline. A, Acellular cleaved A5C1 (w/o cell-L).B, Non-cleaved and cleaved MBVP-laden A5C1 (MBVP-NL and MBVP-L).C, Non-cleaved and cleaved C8D1A-laden A5C1 (C8D1A-NL and C8D1A-L).

**Fig. S3.** A, Larger fields of immunofluorescence staining images for type I collagen (white pseudocolor), focal adhesion protein Talin (red), F-actin (green), and nuclei (DAPI, blue) in the MBVP-laden A5C1 hydrogels cultured under non-cleaved (MBVP-NL) and cleaved (MBVP-L) conditions on day 1 and day 5. B, Larger fields of immunofluorescence staining images for F-actin (green), basement membrane protein laminin (red), and nuclei (DAPI, blue) in the MBVP-laden A5C1 hydrogels cultured under non-cleaved (MBVP-NL) and cleaved (MBVP-L) conditions on day 1 and day 5.

**Fig. S4.** Representative live/dead staining images of C8D1A cells at different time points. Green: Calcein-AM (live cells); red: PI (dead cells). Labels “NL-Dx” refer to day x of non-cleaved culture; “xL-Dy” indicates day y post-cleavage initiated on day x. The white arrows indicate the spreading cells.

Fig. S5. A, Comparison of shear storage moduli among candidate rigid materials and the soft matrix, the candidate rigid materials include A50G50H25 (5 wt% alginate/5 wt% GelMA/2.5 wt% HAMA), A50G50 (5 wt% alginate/5 wt% GelMA), G50H50 (5 wt% GelMA/5 wt% HAMA), A25G50H50 (2.5 wt% alginate/5 wt% GelMA/5 wt% HAMA), A50G100 (5 wt% alginate/10 wt% GelMA), and G100H50 (10 wt% GelMA/5 wt% HAMA), the soft material is A5C1 (0.5 wt% alginate/0.1 wt% collagen). B, Time-dependent viscosity curves of candidate rigid materials. C, Strain amplitude sweep of A50G50, showing its shear-thinning and recovery behavior. D–E: Construction of a soft/rigid hybrid hydrogel tube, where the soft material (0.5wt%Alginate/0.1wt%collagen, A5C1) provides a favorable matrix for cell growth and the rigid material (5wt%Alginate/5wt%GelMA, A50G50) offers mechanical support. (D) Embedded bioprinting of a spring-shaped A50G50 scaffold. (E) Casting of the soft A5C1 matrix to form the vascular lumen. F, A rotary platform enables uniform endothelial adhesion along the lumen wall. G-H, Perfusion system of the in vitro blood-brain barrier model.

**Fig. S6. Mechanical and structural stability of the supportive scaffold after enzymatic cleavage.** A, Representative photographs of rigid A50G50, soft A5C1, and hybrid samples used for compression testing. B, Compressive modulus of rigid, soft, and hybrid samples before and after enzymatic cleavage with 0.2 μg/mL alginate lyase at 37 °C overnight. The rigid A50G50 material showed no significant decrease after cleavage, whereas the soft A5C1 matrix was markedly softened. Hybrid samples retained significantly higher compressive modulus than the soft matrix after cleavage, indicating a sustained load-bearing contribution of the supportive scaffold. C, Representative image of the supportive scaffold after enzymatic cleavage. D, Representative image of the hybrid tubular construct after perfusion culture, treatment with 0.05 μg/mL alginate lyase at 37 °C for 4 h, and subsequent culture to day 5. Data are presented as mean ± SD, n = 3. Statistical significance was determined by Student’s t-tests. ns, not significant; **p < 0.01, ***p < 0.001.

**Fig. S7.** Cross-sectional images of the endothelialized tubular construct containing embedded pericytes and astrocytes during perfusion culture and enzymatic remodeling. Representative bright-field images showed that the lumen remained open at different time points. The intrinsic RFP signal of endothelial cells revealed a ring-like endothelial lining along the luminal boundary, indicating preserved endothelial localization and tubular structural integrity during culture.

Fig.S8. Phase angle (A) and capacitor (B) values were measured using the integrated bioreactor every day. (the graph shows the mean values ± standard deviation in the form of a shaded envelope of the measured values at 1 kHz, n = 4). Seq-Tri indicates the sequential tri-culture group of endothelial cells, pericytes and astrocytes, Syn-Tri indicates the synchronous tri-culture group of endothelial cells, pericytes and astrocytes, Seq-Bi indicates the sequential bi-culture group of endothelial and astrocytes, and Mono indicates the monolayer culture group of endothelial cells.
